# A randomized, double-blind trial comparing the effect of two blood pressure targets on global brain metabolism after out-of-hospital cardiac arrest

**DOI:** 10.1186/s13054-023-04376-y

**Published:** 2023-02-24

**Authors:** Simon Mølstrøm, Troels Halfeld Nielsen, Carl-Henrik Nordstrøm, Axel Forsse, Søren Møller, Søren Venø, Dmitry Mamaev, Tomas Tencer, Ásta Theódórsdóttir, Thomas Krøigård, Jacob Møller, Christian Hassager, Jesper Kjærgaard, Henrik Schmidt, Palle Toft

**Affiliations:** 1 Department of Anesthesiology and Intensive Care, Odense University Hospital, J. B. Winsløws Vej 4, 5000 Odense, Denmark; 2 Department of Neurosurgery, Odense University Hospital, Odense, Denmark; 3 OPEN, Open Patient Data Explorative Network, Odense University Hospital, Odense, Denmark; 4 Department of Clinical Research, University of Southern Denmark, Odense, Denmark; 5 Department of Neurology, Odense University Hospital, Odense, Denmark; 6 The Heart Centre, Copenhagen University Hospital, Copenhagen, Denmark; 7 Department of Neurosurgery, Copenhagen University Hospital, Copenhagen, Denmark; 8 Department of Cardiology, Odense University Hospital, Odense, Denmark; 9 Department of Clinical Medicine, University of Southern, Odense, Denmark

**Keywords:** Out-of-hospital cardiac arrest, Brain injury, Arterial pressure, Jugular bulb microdialysis, Cerebral metabolism

## Abstract

**Purpose:**

This study aimed to assess the effect of different blood pressure levels on global cerebral metabolism in comatose patients resuscitated from out-of-hospital cardiac arrest (OHCA).

**Methods:**

In a double-blinded trial, we randomly assigned 60 comatose patients following OHCA to low (63 mmHg) or high (77 mmHg) mean arterial blood pressure (MAP). The trial was a sub-study in the Blood Pressure and Oxygenation Targets after Out-of-Hospital Cardiac Arrest-trial (BOX). Global cerebral metabolism utilizing jugular bulb microdialysis (JBM) and cerebral oxygenation (rSO_2_) was monitored continuously for 96 h. The lactate-to-pyruvate (LP) ratio is a marker of cellular redox status and increases during deficient oxygen delivery (ischemia, hypoxia) and mitochondrial dysfunction. The primary outcome was to compare time-averaged means of cerebral energy metabolites between MAP groups during post-resuscitation care. Secondary outcomes included metabolic patterns of cerebral ischemia, rSO_2_, plasma neuron-specific enolase level at 48 h and neurological outcome at hospital discharge (cerebral performance category).

**Results:**

We found a clear separation in MAP between the groups (15 mmHg, *p* < 0.001). Cerebral biochemical variables were not significantly different between MAP groups (LPR low MAP 19 (16–31) vs. high MAP 23 (16–33), *p* = 0.64). However, the LP ratio remained high (> 16) in both groups during the first 30 h. During the first 24 h, cerebral lactate > 2.5 mM, pyruvate levels > 110 µM, LP ratio > 30, and glycerol > 260 µM were highly predictive for poor neurological outcome and death with AUC 0.80. The median (IQR) rSO_2_ during the first 48 h was 69.5% (62.0–75.0%) in the low MAP group and 69.0% (61.3–75.5%) in the high MAP group, *p* = 0.16.

**Conclusions:**

Among comatose patients resuscitated from OHCA, targeting a higher MAP 180 min after ROSC did not significantly improve cerebral energy metabolism within 96 h of post-resuscitation care. Patients with a poor clinical outcome exhibited significantly worse biochemical patterns, probably illustrating that insufficient tissue oxygenation and recirculation during the initial hours after ROSC were essential factors determining neurological outcome.

**Supplementary Information:**

The online version contains supplementary material available at 10.1186/s13054-023-04376-y.

## Introduction

Survival rates remain around 50% for comatose patients treated in the Intensive Care Unit (ICU) after out-of-hospital cardiac arrest (OHCA) [1–3]. ICU mortality is mainly caused by hypoxic/ischemic brain injury related to delayed cerebral hypoperfusion, ischemia–reperfusion injury, mitochondrial dysfunction, and impaired autoregulation [4–10]. Post-ischemic neuronal death occurs in the hours and days after ROSC, which may open a therapeutic window to improve outcomes. Unfortunately, no technique has been available for routine assessment of cerebral energy metabolism following OHCA. Recently, our group has in experimental studies shown that after induced global cerebral ischemia mitochondrial oxidative energy metabolism can be evaluated online by performing microdialysis of the draining venous blood [11, 12]. The technique has been assessed in explorative clinical studies by performing jugular bulb microdialysis (JBM) [13]. Microdialysis provides the most direct means to monitor cerebral "energy failure" in real time. Conventionally, the lactate-to-pyruvate (LP) ratio is a marker of cellular redox status, and increased levels are associated with unfavorable outcome in neurocritical care [14–17]. The ratio increases mainly during deficient oxygen delivery (ischemia, hypoxia) and mitochondrial dysfunction [18–22].

Adequate mean arterial blood pressure (MAP) is vital to maintain cerebral blood flow (CBF) at a level sufficient for preserving oxidative energy metabolism. Cerebral autoregulation is impaired or right-shifted in approximately 30–50% of patients following cardiac arrest [5, 7, 23]. Consequently, hypotension after cardiac arrest may result in cerebral hypoperfusion and oxygenation, worsening brain injury. However, randomized controlled trials studying specific MAP targets in post-resuscitation care have failed to show any signs of benefit on surrogate outcomes [24, 25]. No clinical studies have shown that targeted MAP using transcranial Doppler, cerebral oxygen saturation (rSO_2_), and pressure reactivity index influence neurological outcome [5, 26–28].

This randomized trial was designed to compare the effect of two levels of MAP on global cerebral energy metabolism in comatose patients resuscitated from OHCA. Therefore, we tested the hypothesis that higher MAP improves CBF, cerebral oxygenation and oxidative metabolism by lowering the LP ratio, during post-resuscitation care compared with lower MAP.

## Methods

### Trial design

The trial was a sub-study in the Blood Pressure and Oxygenation Targets after Out-of-Hospital Cardiac Arrest-trial (BOX), see Supplementary Methods [29–31]. The design of the double-blinded, randomized clinical trial and the statistical analysis plan have been published previously [32].

According to national requirements and the principles of the Declaration of Helsinki, informed consent was obtained from next of kin and one independent medical doctor not involved with the trial. In addition, informed consent was obtained from patients who regained appropriate neurological function for independent decision-making. The Danish Regional Committee on Health and Research Ethics and Danish Data Protection Agency approved the protocol (S-20150173 HLP). The study was registered at ClinicalTrials.gov, identifier: NCT03095742 (30/03/2017).

### Patients

This sub-study included patients in a tertiary heart center, Odense University Hospital, Denmark. Inclusion and exclusion criteria have been published elsewhere [32]. In brief, patients aged ≥ 18 years with OHCA of presumed cardiac cause, sustained return of spontaneous circulation and score ≤ 8 on the Glasgow Coma Scale (GCS) were enrolled. The main exclusion criteria were an interval from ROSC to randomization > 240 min, and unwitnessed asystole. For this sub-study only patients randomized to liberal oxygen targeting PaO2 13–14 kpa were enrolled [31]. A complete list of all inclusion and exclusion criteria is provided in the Supplementary Appendix.

### Randomization and blinding

Patients were randomly assigned in a 1:1 ratio to low or high MAP using a web-based system involving dynamic permuted block sizes. The MAP intervention was double-blinded (see intervention). The physicians taking care of trial patients and assessing neurological prognosis were not blinded to bedside JBM and cerebral saturation. The neurophysiologist analyzing the EEGs and evaluating neurologic outcomes was unaware of the trial-group assignments and microdialysis data.

### Intervention

The MAP intervention commenced at randomization and continued as long as the patients needed invasive blood pressure measurements. Patients were randomly monitored with a module (Phillips M1006B) offset to – 10% or a module offset to + 10%. Targeting a MAP of 70 mmHg during treatment in both groups caused a blinded comparison of approximately 63 and 77 mmHg, a 20% separation (Supplementary Methods). A randomized clinical study has validated the method for double-blinded comparison of MAP targets in the ICU setting [33].

A protocol provided a recommendation for targeting a MAP of 70 mmHg in a three-stage approach: volume resuscitation to a central venous pressure of 10 mmHg, norepinephrine infusion, and the addition of a dopamine infusion for a maximal dose of 10 μg per kilogram of body weight per minute, if needed.

### Post-resuscitation procedure

Targeted temperature management (TTM) commenced at ICU admission targeting 36.0 °C for 24 h, followed by rewarming at a rate of 0.5 °C/h. Sedation was mandated in both groups during the TTM period. Mechanical ventilation was adjusted to ensure normocapnia (PaCO_2_ of 4.5 – 6.0 kPa), and oxygenation was kept in the range of 13–14 kPa. The blood glucose level was strictly maintained between 6 and 10 mmol/l. Details regarding clinical procedures and administered drugs are provided in the Supplementary Methods. Study data were collected and managed using REDCap electronic data capture tools hosted at Open Patient Data Explorative Network [34].

### Neuromonitoring

JBM commenced after ICU admission and continued for 96 h or until arousal utilizing identical techniques as previously published (Additional file 1: Figs. S2 and S3) [13, 35, 36]. The perfusates obtained from JBM were analyzed hourly for glucose, lactate, pyruvate, glycerol, and glutamate, utilizing conventional enzymatic techniques [13]. As the pilot study showed a strong correlation between systemic blood lactate and *microdialysis arterial lactate*, the former was used as a reference in the present study (Supplementary Methods). The accurate positioning of the jugular bulb catheter tip was verified on CT scans (see illustrations in Additional file 1: Figs. S2–S3).

Bilateral, regional cerebral oxygen saturation (rSO_2_) was recorded continuously every hour for 96 h or until awakening (Somanetics INVOS Cerebral Oximeter system) with a predefined desaturation threshold of 50% [37]. The hourly rSO_2_ values of the left and right frontal sensors were averaged and used in the analysis. The clinicians did not change clinical practice based on either bedside rSO_2_ or JBM monitoring.

The biomarker of neurological damage, plasma neuron-specific enolase (NSE) level at 48 h, was measured by electrochemiluminescence and by a COBAS analyzer system (Roche Diagnostics) [38].

### JBM reference values and classification of ischemia

The definition of normal microdialysate values (lactate, pyruvate, glucose, glycerol, glutamate, LP ratio) in human jugular venous blood was based on JBM reference values obtained in anesthetized patients undergoing elective cardiac bypass surgery [35]. These preoperative reference values were used to define the pathological thresholds for each variable (mean ± 2SD) indicated in Figs. 2 and 3.

Definitions of the biochemical patterns for cerebral ischemia and mitochondrial dysfunction were based on principles obtained from intracerebral microdialysis [22, 39]. In this study, LP ratio > 16 at pyruvate < 70 µM was classified as a pattern indicating ischemia, and LP ratio > 16 at pyruvate > 70 µM demonstrated mitochondrial dysfunction [35, 39].

### Neurologic prognostication

Active treatment continued until 72 h after TTM, except for patients with status myoclonus, brain death, or refractory shock with multiple organ failure. Patients who remained comatose despite cessation of sedation were evaluated by combining neurologic examination, EEG, somatosensory-evoked potential (SSEP), biomarkers (NSE) and brain CT. As previously described, EEGs were classified as highly malignant, malignant, or benign [40]. Assessment of neurological outcome was performed by a multidisciplinary team in accordance with guidelines (Supplementary Methods) [41].

### Outcome

The primary outcome was the difference between time-averaged means of microdialysis parameters and LP ratio (intervals of 12 h) between low vs high MAP group within 96 h of post-resuscitation care. Secondary outcomes of clinical interest were to compare (i) variables reflecting cerebral energy metabolism and patterns of ischemia and mitochondrial dysfunction in patients with unfavorable and favorable neurological outcome [39], (ii) cerebral oxygenation (rSO_2_) between MAP groups, (iii) biochemical variables in relation to critical clinical episodes such as EEG-verified seizures and hypotension and (iv) neurological outcome at hospital discharge and 90 days after OHCA. Additional secondary outcomes were association between JBM markers, neurological outcome and brain injury defined by NSE levels at 48 h. As the study was planned and powered for the primary outcome, the secondary outcomes should be considered exploratory and interpreted with caution.

Neurological outcome was assessed at hospital discharge and 90 days after OHCA according to the Cerebral Performance Category (CPC) scale [42, 43]: CPC scores of 1–2 are considered 'favorable' outcomes, and a CPC 3–5 ‘unfavorable’ outcomes.

The adverse events included in this report are bleeding, infection, arrhythmia, and seizures as well as complications related to the MD technique.

### Statistical methods

We estimated that a sample of 46 patients would provide 90% power to detect a relative LP ratio reduction of 35% in the high MAP group, compared with the low MAP group, using a patient-to-patient variation with standard deviation (SD) of 15 [32, 35]. To allow for possible higher patient-to-patient variation and deviations from normality, a sample size of 60 was chosen.

The mean between-group difference in blood pressure during the intervention period was calculated by mixed effects linear regression. Dynamic changes of MD variables were compared between treatment groups by linear mixed effects linear regression (Supplementary Methods).

Time-averaged means of MD parameters in intervals of 12 h ensured statistical robustness in contrast to point values. Note that all hourly measurements were included in the mixed models, and only aggregated in the parametrization of the model, not in the measurements themselves.

The mixed model fitting procedure handled missing values, assumed to be missing completely at random. Missing MD data rates were less than 5% and imputation where not used. Missing measurements were taken into account by the maximum likelihood estimation of the mixed models. Associations between MD variables, episodes of hypotension, EEG-verified seizures, cerebral desaturation, and neurological outcome were assessed with chi-square-test and logistic regression. Statistical comparison of episodes with ischemia and cerebral desaturation between outcome groups was performed using Mann–Whitney *U*-test. Associations between peak values of MD variables and outcome groups were explored by applying a *t*-test. A *p*-value < 0.05 was considered statistically significant. Statistical analyses were performed using Stata V.16 (Stata Corporation, College Station, Texas, USA).

## Results

### Patients

A flowchart of the patient enrollment and group allocation is presented in the Supplementary Appendix (Additional file 1: Fig. S1). Recruitment began on 22 October 2017 and was completed by 19 May 2020. Sixty unconscious patients with sustained ROSC admitted to the intensive care unit following OHCA completed the trial and were included in the analysis. The two MAP groups had similar baseline characteristics (Table 1). Details are provided in Additional file 1: Tables S1–S3, S7 and Supplementary Results.

**Table 1:** Baseline characteristics according to MAP intervention

Characteristic	Low MAP *N* = 30	High MAP *N* = 30
Demographic characteristics		
Age–years	69 ± 12	63 ± 14
Male sex–no. (%)	28 (93)	24 (80)
Medical history–no. (%)		
Chronic heart failure	8 (27)	3 (10)
Ischemic heart disease	11 (37)	7 (23)
Arterial hypertension	20 (67)	14 (47)
Previous stroke	3 (10)	0 (0)
Diabetes mellitus	7 (23)	4 (13)
Previous percutaneous coronary intervention^a^	6 (20)	5 (17)
Neurological function before cardiac arrest		
Normal, CPC score 1*	26 (87)	29 (97)
Some disability, CPC score 2	4 (13)	1 (3)
Characteristics of the cardiac arrest		
Witnessed cardiac arrest–no. (%)^b^	27 (90)	26 (87)
Bystander performed CPR–no. (%)	26 (87)	29 (97)
First monitored rhythm–no. (%)		
Shockable rhythm	22 (73)	22 (73)
Time from cardiac arrest to event–min		
Start of basic life support, median (IQR)	2 [1–5]	1 [1–3]
Start of advanced life support, median (IQR) Return of spontaneous circulation, median (IQR)	8 [5–10] 16 [10–23]	10 [6–16] 20 [12–32]
Clinical characteristics on admission		
First measured body temperature–°C	35.5 ± 1.0	35.1 ± 1.3
Glascow Coma Scale score §, median (IQR)	3 [3–3]	3 [3–3]
Pupillary reflex present–no. (%)	17 (57)	19 (63)
Serum pH^c^ Serum lactate–mmol/liter	7.26 ± 0.12 5.1 ± 4.1	7.23 ± 0.15 5.2 ± 4.3
Shock–no. (%) ¶	11 (36)	8 (26)
ST-segment elevation myocardial infarction–no. (%)	8 (27)	18 (60)

Plus-minus values are means ± SD. Abbreviations: CPC, Cerebral Performance Category; AMI, acute myocardial infarction; CPR, cardiopulmonary resuscitation; IQR, interquartile range. * CPC score: 1, alert, able to work and lead a normal life; 2, moderate cerebral disability and sufficient cerebral function for part-time work; 3, severe cerebral disability, dependent on others, and impaired brain function; 4, coma and vegetative state; 5, dead or certified brain dead. § Scores on the Glasgow Coma Scale range from 3 to 15, with lower scores indicating reduced levels of consciousness. ¶ Shock was defined as a systolic blood pressure < 90 mmHg for more than 30 min or end-organ hypoperfusion (cool extremities, confusion, urine output < 0.5 ml/kg per hour, lactate > 2.5 mmol/l)

^a^Data missing for one patients

^b^Data missing for one patients

^c^Data missing for two patients

### Intervention

**Fig. 1:**
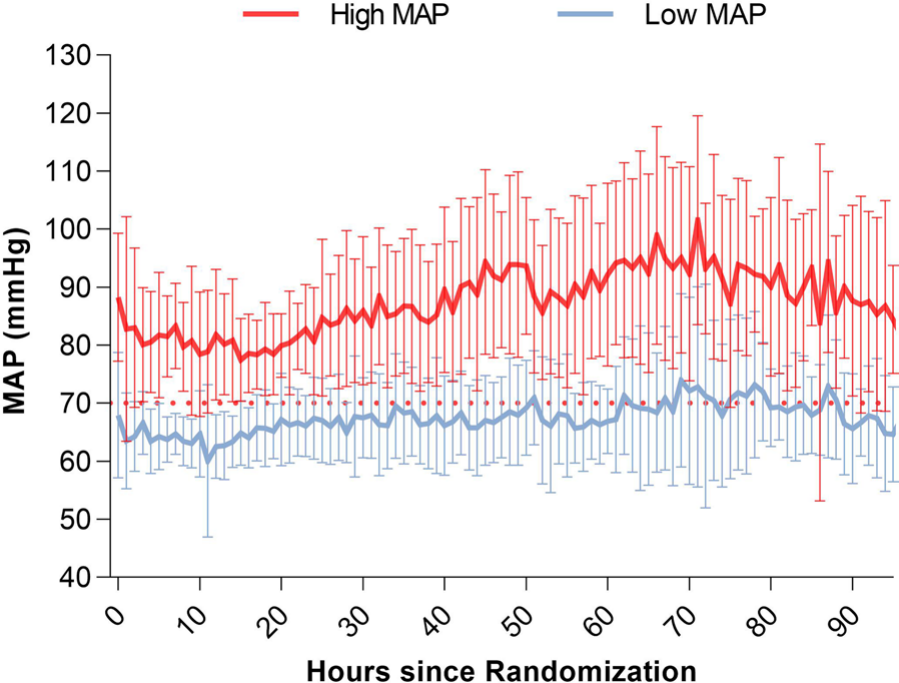
Mean arterial pressure during the intervention period. The MAP curves show the means, and the bars indicate ± 2 SD (95% of the observations are within the error bars). A clear separation in MAP between the groups (mean difference: 15 mmHg, *p* < 0.0001) was observed. The dotted red line indicates the target MAP of 70 mmHg. The median time from return of spontaneous circulation to randomization in the trial was 180 min

The median time from ROSC to randomization in the trial was 180 [120–210] min. There was a clear separation in MAP between the groups (mean difference: 15 mmHg ([CI], 13.1 to 17.3), p < 0.0001) (Fig. 1). The average MAP values during the first 24 h were marginally higher than targeted in both groups (high MAP: 80 mmHg and low MAP: 65 mmHg). In the low MAP group, the norepinephrine dose and Vasoactive-Inotropic Score (VIS) was significantly higher (*p* = 0.0001) during the first 24 h after admission (Additional file 1: Fig. S4 and Table S1).

Additional regulators of cerebral blood flow remained within therapeutic range with no difference in cardiac index, PaO_2_ and PaCO_2_ at any time points between MAP groups (Additional file 1: Table S1).

### Outcome

#### Jugular bulb microdialysis

The median time from ROSC to microdialysis monitoring was 260 min in the low and high MAP groups with monitoring durations of 65 [47–91] and 63 [45–89] hours, respectively (Additional file 1: Table S3). Median time from MAP intervention to MD monitoring was 76 min.

#### Association between cerebral energy metabolism and MAP

**Fig. 2:**
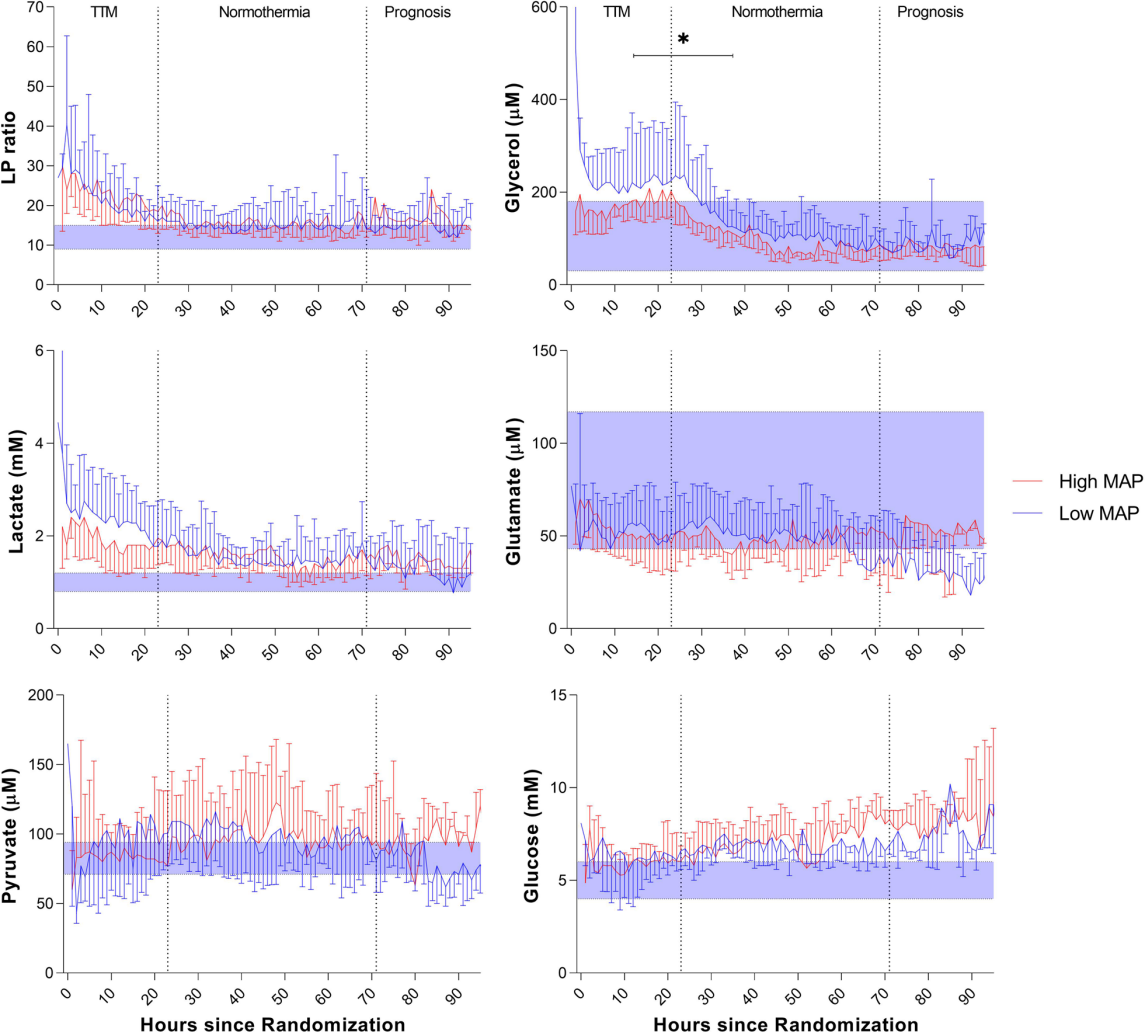
Data are expressed as median (interquartile range). LP ratio: lactate/pyruvate ratio. Jugular bulb microdialysis variables in the study groups (*n* = 60) during MAP intervention. The median time from return of spontaneous circulation to microdialysis monitoring in the trial was 260 min. * The difference between time-averaged means (in intervals of 12 h) of the variables was insignificant during post-resuscitation care except for glycerol when using mixed effects models. The graphs display normal reference values (shaded area) for each JBM variable: LP ratio (12 ± 3); Lactate (1.0 ± 0.2 mM); Pyruvate (82 ± 11 µM); Glycerol (105 ± 75 µM); Glutamate (80 ± 37 µM); Glucose (5.0 ± 1.0 mM) [35]

No significant difference in microdialysis values between the two MAP groups was observed except for glycerol in the early post-resuscitation phase (12–36 h, *p* < 0.001) (Fig. 2). When adjusted for MAP differences, a significant positive correlation between norepinephrine dose and glycerol was observed within the first 36 h. The LP ratio remained high (> 16) in both groups during the first 30 h (Fig. 2 and Additional file 1: Table S5). Irrespective of MAP allocation, 75% of patients exhibited pronounced secondary ischemia (Additional file 1: Table S3 and Supplementary Results).

#### Association between cerebral energy metabolism and neurological outcome

**Fig. 3:**
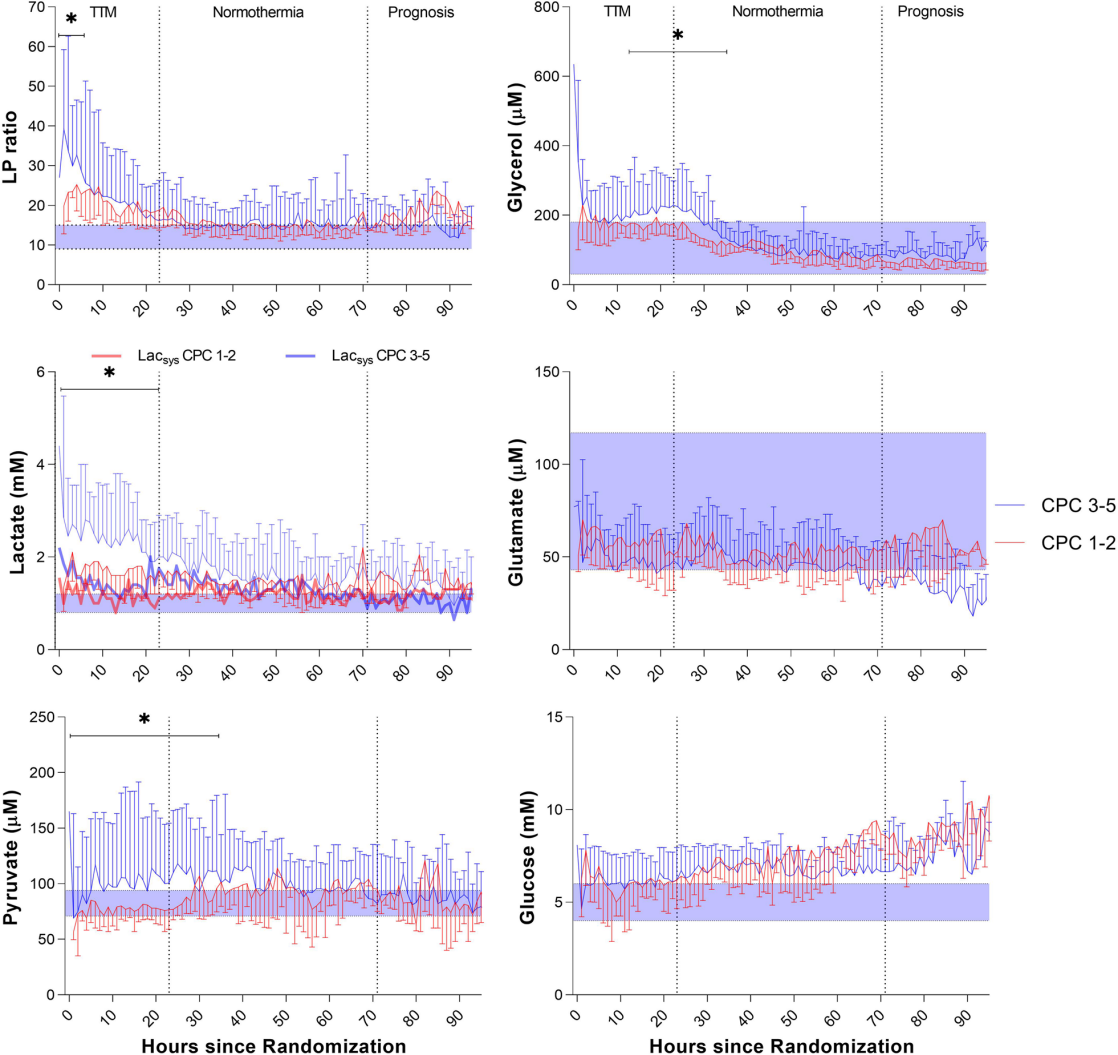
Median (IQR). Jugular bulb microdialysis parameters during the intervention period in patients discharged with unfavorable outcome (black line) (CPC 3–5, *n* = 37) compared to patients with favorable outcome (red line) (CPC 1–2, *n* = 23). The median time from return of spontaneous circulation to microdialysis monitoring in the trial was 257 min. * The difference between time-averaged means of LP ratio (interval of 6 h) and lactate, pyruvate, and glycerol (intervals of 12 h) was significant during post-resuscitation care when using mixed effects models. JBM variables were not stratified for MAP group. In patients with unfavorable outcome, a significant difference between jugular bulb lactate and systemic lactate (Lac_sys_) was observed for the entire period. During the first 24 h, systemic lactate was significantly higher in the CPC 3–5 group compared to CPC 1–2 group. The graphs display normal reference values (shaded area) for each JBM variable: LP ratio (12 ± 3); Lactate (1.0 ± 0.2 mM); Pyruvate (82 ± 11 µM); Glycerol (105 ± 75 µM); Glutamate (80 ± 37 µM); Glucose (5.0 ± 1.0 mM) [35]

Figure 3 compares changes in biochemical variables for outcome groups CPC 1–2 and CPC 3–5. During the first 24 h, JBM lactate was significantly higher in the CPC 3–5 group (mean level > 2.3 mM and peak level > 16 mM). JBM pyruvate was considerably higher in the poor outcome group during the initial 36 h, and LP ratio was significantly higher during the first six hours (Fig. 3 and Additional file 1: Table S6). Significantly increased glycerol levels were obtained during the period 12–36 h after start of JBM (CPC 3–5). During the first 24 h, cerebral lactate > 2.0 mM, pyruvate levels > 100 µM, LP ratio > 20, and glycerol > 200 µM predicted poor outcome (CPC 3–5) with AUC 0.73. A significant difference between jugular bulb lactate and systemic lactate in patients with unfavorable outcomes was observed during the first 36 h. Baseline characteristics for outcome groups are listed in Table S4. No significant difference between CPC groups related to the extent of cerebral ischemia and mitochondrial dysfunction was observed (Additional file 1: Appendix).

Non-survivors exhibited significantly increased levels of lactate, pyruvate, and glycerol (*p* < 0.0001) during the total MD monitoring period compared to survivors. During the first 24 h, cerebral lactate > 2.5 mM, pyruvate levels > 110 µM, LP ratio > 30, and glycerol > 260 µM predicted death with AUC 0.80, obtaining a sensitivity of 70% (46–87%) and a specificity of 85% (70–93%).

At 48 h, the median NSE (*n* = 39) level was significant lower in the CPC 1–2 group compared to the CPC 3–5 group (11 [7–13] μg per liter vs. 22 [11–40] μg per liter, *p* = 0.006). Irrespective of neurological outcome, correlation between JBM variables such as LP ratio and lactate showed no significantly correlation to median neuron-specific enolase levels at 48 h. However, in patients with poor outcome a relative high correlation for cerebral lactate and NSE levels (*n* = 25) was observed, *R* = 0.73.

#### Regional cerebral desaturation

**Fig. 4:**
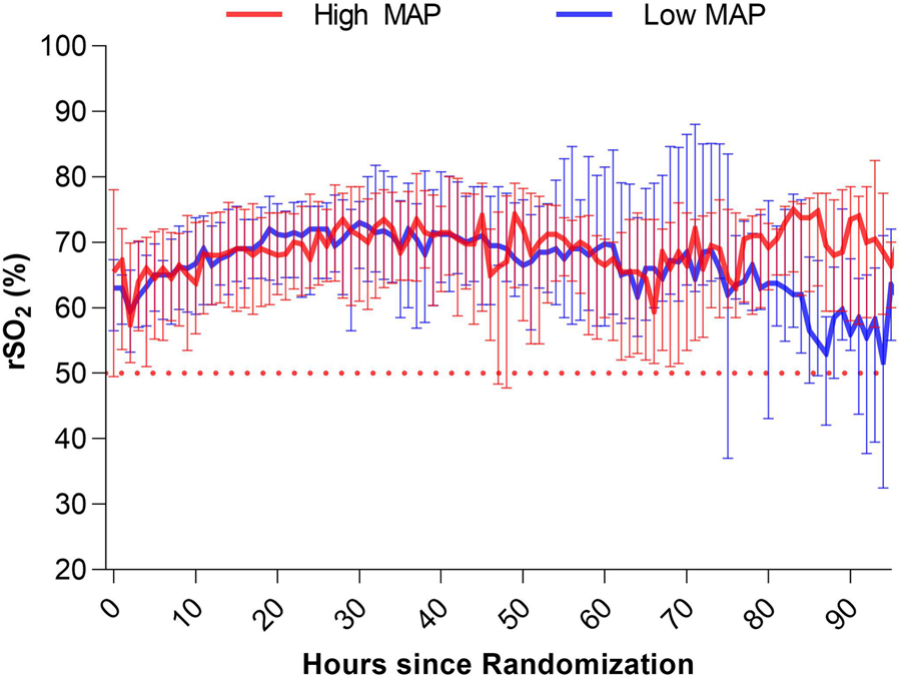
Median (IQR). Regional cerebral oxygen saturation (rSO_2_) during MAP intervention in the study groups (*n* = 60). No significant differences between MAP groups were observed. Data not stratified for neurological outcome. The dotted red line indicates cerebral desaturation threshold rSO_2_ < 50%

During MAP intervention, no significant differences in rSO_2_ between MAP groups were observed at any time point using mixed effects models (Fig. 4 and Additional file 1: Table S3). The median (IQR) rSO_2_ during the first 48 h was 69.5% (62.0–75.0%) in the low MAP group and 69.0% (61.3–75.5%) in the high MAP group, *p* = 0.16. Irrespective of chronic hypertension, no significant differences in rSO_2_ between MAP groups were observed at any time point using mixed effects models (Additional file 1: Figs. S6-S7). Data were not stratified for neurological outcome since similar non-significant differences between outcome groups were observed (Additional file 1: Fig. S5). No correlations between JBM-verified ischemic periods and cerebral desaturation were observed.

#### JBM variables in relation to critical episodes

JBM levels in patients with EEG-verified epileptiform activity were not significantly different from other patients, except for significantly higher pyruvate levels during the first 48 h (*p* = 0.001). Jugular bulb lactate, LP ratio, and glycerol correlated negatively to the corresponding MAP in all patients. For each one-unit increase in MAP (mmHg), cerebral lactate changed by − 0.010 mM (95% CI − 0.014; − 0.006, *p* < 0.001), LP ratio − 0.16 (95% CI − 0.28; − 0.04, *p* = 0.01) and glycerol by − 1.5 µM (95% CI − 1.81; − 1.17, *p* < 0.001). Patients with preexisting hypertension responded even more for each one-unit increase in MAP; lactate − 0.014 mM, (95% CI − 0.019; − 0.009, *p* < 0.001), LP − 0.34 (95% CI − 0.51; − 0.18, *p* < 0.001).

#### Neurological outcome

At hospital discharge 22 of 30 patients (73%) in the low MAP group and 15/30 (50%) in the high MAP group had a poor functional outcome CPC 3–5. Within 90 days, 12 of 30 patients (40%) in the low MAP group and 8 of 30 patients (27%) in the high MAP group had died. Overall mortality during hospital stay was 33% due primarily to severe anoxic brain injury and withdrawal of life-sustaining therapy. At 48 h, the median NSE level was 15 μg per liter in the low MAP group and 12 μg per liter in the high MAP group, *p* = 0.92. Detailed results for outcomes are given in Additional file 1: Table S3.

### Adverse events

No significant differences were found in the percentages of patients with serious adverse events, including sepsis, arrhythmia, seizures and bleeding (Additional file 1: Table S3). Complications related to the MD technique were not observed.

## Discussion

This double-blinded randomized trial compared two blood pressure targets in comatose patients resuscitated from OHCA. Targeting a higher MAP (77 vs. 63 mmHg) did not significantly improve cerebral energy metabolism within 96 h of post-resuscitation care. No secondary outcomes, including cerebral oxygenation (rSO_2_), NSE levels, metabolic patterns of ischemia, or neurological outcome, were significantly affected by higher MAP.

Bedside monitoring of global cerebral energy metabolism is a novel technique. It is based on experimental studies in pigs showing that microdialysis of the draining venous cerebral blood gives semi-quantitative information of cerebral interstitial levels of lactate, pyruvate and LP ratio at cerebral ischemia [11, 12]. The technique requires that brain ischemia and the perturbation of energy metabolism can be assumed to be global and has been evaluated in explorative clinical studies during cardiopulmonary bypass in open heart surgery and after OHCA [13, 35]. The interpretations of observed changes in the levels of the biochemical variables after ROSC are based on our previous experimental studies of transient global cerebral ischemia evaluated from whole brain tissue homogenates as well as intracerebral microdialysis. These studies were summarized in a recent review [39].

Secondary brain injury seems to occur in the early period after OHCA and is driven by complex pathophysiological mechanisms such as reperfusion injury with mitochondrial dysfunction, impaired autoregulation and delayed hypoperfusion during the first 12 to 24 h after cardiac arrest [7, 23, 44, 45]. Observational data suggests that these processes may be may be exacerbated by hypotension [46].

Despite a clinically significant separation between MAP groups (Fig. 1) of approximately 15 mmHg in the present study, we found no significant between-group differences in the levels of lactate, pyruvate, or the LP ratio during the 96 h study period (Fig. 2). Furthermore, a higher MAP did not decrease NSE levels at 48 h, indicating an insignificant therapeutic effect and/or difference in brain injury/hypoxia between MAP groups. The results agree with the main BOX trial (MAP difference 10.7 mmHg), showing no significantly different percentages of patients dying, having severe disability (CPC 3–4) or biomarkers of neurologic brain injury [30]. Furthermore, other prospective, multi-center, randomized trials showed that targeting a specific MAP range (170 min after ROSC) after transfer to the ICU did not affect surrogate outcomes [24, 25].

Compromised cerebral metabolism and brain hypoxia probably depend on several factors. Despite optimized MAP and oxygen delivery, diffusion limitations of oxygen [47], mitochondrial dysfunction and blood–brain barrier breakdown with secondary edema or cell death may exacerbate secondary injury [48]. A sub-set of patients with hypoxic/ischemic brain injury are perfusion dependent with intact oxygen diffusion, which may benefit from an augmented and individualized MAP. Targeting a higher mean arterial pressure in the post-resuscitation period may be warranted in patients with preexisting hypertension [23]. Nevertheless, subgroup analysis of the primary outcome in the BOX trial suggested no benefit of a higher blood pressure target in patients with known hypertension.

Interestingly, JBM revealed that jugular bulb lactate, LP ratio, and glycerol correlated negatively to the corresponding MAP in all patients. Moreover, we were able to discriminate specific subgroups (preexisting hypertension), benefiting more from an augmented MAP on cerebral metabolism.

Cerebral reperfusion after OHCA is complex and there is a lack of data regarding the first hours after ROSC. In both MAP groups, a majority exhibited severe disturbance in cerebral energy metabolism with markedly increased LP ratio up to 20 h after the start of JBM (Fig. 2). As the median delay of JBM was 260 min after ROSC, normalization of LP ratio was not observed until 1400–1600 min after ROSC. A similar long-lasting perturbation was described using intracerebral MD after CA [49].

In experimental studies, near-normalization of LP ratio is obtained 60–120 min after recirculation in animals exposed to 30 min of complete cerebral ischemia [50, 51]. Mitochondrial aerobic activity will be almost normalized within this time limit, provided adequate cerebral blood flow is obtained immediately with recirculation [52]. A porcine cardiac arrest model also described normalized LP ratio 30–60 min after ROSC [53]. The discrepancy between previous experimental data and our clinical findings supports the hypothesis that initial cerebral reperfusion in most patients is insufficient to restore cytoplasmic redox state. Furthermore, recent clinical studies described brain tissue hypoxia and associated active release of biomarkers of neuronal injury in about half of comatose patients with brain injury (13–40 h post-arrest) [47, 54].

Though the LP ratio may return to a near-normal level after transient cerebral ischemia, the insult often causes lasting mitochondrial dysfunction [55, 56]. Consequently, the tissue level of lactate remains high and is paralleled by a marked increase in pyruvate [22, 50]. Furthermore, neuronal function is also influenced by transmission failure and delayed neuronal death [57, 58].

Although JBM did not start until about 260 min after ROSC, significant differences were obtained between the two outcome groups (CPC 1–2 vs. CPC 3–5) for variables related to oxidative energy metabolism (lactate, pyruvate, LP ratio) (Fig. 3). The marked increase in LP ratio in the CPC 3–5 group during the first 12 h of JBM indicates that tissue oxygenation and recirculation immediately after ROSC was worse in this group than in the CPC 1–2 group.

In the CPC 3–5 group, lactate and pyruvate remained higher than in the CPC 1–2 group for up to 60 h (Fig. 3) indicating mitochondrial dysfunction secondary to a more severe initial ischemic insult. During the first 24 h, cerebral lactate > 2.5 mM, pyruvate levels > 110 µM, LP ratio > 30, and glycerol > 260 µM was highly predictive for poor neurological outcome and death with AUC 0.80. NSE levels were significantly higher in the CPC 3–5 group, and a relative high correlation for cerebral lactate and NSE levels was observed, *R* = 0.73.

Identifying patients bedside suffering from severe mitochondrial dysfunction using JBM might optimize prognostication and individualize (improving oxygen utilization) the treatment of post-cardiac arrest patients and improve outcome.

Moreover, JBM revealed significantly higher pyruvate levels with EEG-verified epileptiform activity during the first 48 h (CPC 3–5).

Glycerol was significantly increased in the low MAP group during a prolonged period (Fig. 2). An increase in intracerebral glycerol is considered a marker of the degradation of phospholipids in cellular membranes [59]. The increase in glycerol in the low MAP group and the tendencies to higher lactate and lower pyruvate during the initial 20 h of JBM might indicate that lower MAP influence cerebral energy metabolism also 3–4 h after ROSC. However, lipolytic activity in adipose tissue is enhanced by adrenergic mechanisms mediated via beta-adrenoreceptors [60–62]. Accordingly, the noted increase in glycerol may be influenced by the higher VIS score in the low MAP group.

No difference in rSO_2_ was observed between the two MAP groups (Fig. 4) or the outcome groups (Additional file 1: Fig. S5), which underlines that JBM yields information not obtained with other techniques. These results underscore the limitations of rSO_2_-monitoring as a surrogate for cerebral blood flow [25, 26, 63]. Moreover, studies have shown that INVOS has poor agreement of direct tissue measurements of brain oxygenation in post-arrest patients and correlates poorly with direct measures of cerebral autoregulation and blood flow [5, 64, 65].

On the other side the non-significant difference between MAP groups could reflect an intact cerebral autoregulation. However, irrespective of chronic hypertension, no significant differences in rSO_2_ between MAP groups were observed at any time point, indicating that disturbed autoregulation is probably not a major issue, see Additional file 1: Figs. S6-S7.

## Limitations

Our study has several limitations. The assumption for the main study (BOX) 2 × 2 factorial design was that the effects of the MAP and Oxygen interventions were independent (no interaction) in relation to the primary outcome (death or CPC 3–4). However, central factors influencing cerebral metabolism are mainly, e.g., MAP, carbon dioxide and oxygen levels. These main regulators of cerebral metabolism have the potential of important interaction, which justified the exclusion of the “restrictive” oxygen group.

The study is mainly limited by the fact that, due to established clinical routines, it was impossible to start JBM until about 3–4 h after ROSC. The clinical impact of the JBM technique should be re-evaluated when the JBM catheter is positioned within 60 min after ROSC. The blinded MAP intervention was limited to a 20% separation, because the module manufacturer did not support a larger modification without changing the module software. Based on the main BOX trial, we expected significantly higher vasopressor-doses in the high MAP group [30]. Still, the opposite was observed during the first 24 h caused by a higher percentage of shock and organ hypoperfusion in the low MAP group.

Given the known significant limitations of INVOS monitoring to estimate meaningful cerebrovascular physiology, we were not able to state that a higher MAP did not improve brain oxygenation.

Interpretation of the biochemical patterns is based on experiences from intracerebral microdialysis. Further investigations regarding the relationship between global brain 18-FDG-PET (cerebral metabolic rate of glucose) and levels of biochemical variables in cerebral tissue and the draining venous blood are necessary. We did not measure CBF during the study periods to demonstrate coherent findings in cerebral blood flow and JBM. However, our results reflect the complex pathophysiologic mechanisms underpinning secondary brain injury, and combined JBM, CBF measurements (e.g., brain ^15^O-H_2_O-PET CT, Xenon-enhanced CT), and monitoring of mitochondrial function (^18^F-BCPP-EF PET-CT) might provide additional future insights into the dynamics of pathogenic mechanisms [66]. Currently, we are recruiting patients in an exploratory trial: Combined Microdialysis and FDG-PET Study for Detection of Brain Injury After Cardiac Arrest (COMA-PROTECT), ClinicalTrials.gov Identifier: NCT04774055.

## Conclusions

In comatose patients resuscitated from OHCA, targeting a higher MAP 180 min after ROSC did not significantly improve cerebral energy metabolism within 96 h of post-resuscitation care. Patients with a poor clinical outcome exhibited significantly worse biochemical patterns documenting that inadequate tissue oxygenation and reperfusion instantly after ROSC is an essential factor determining neurological restoration.

## Supplementary Information

**Additional file 1**. Supplementary appendix.

## Data Availability

Deidentified data are available for sharing. The dataset analyzed in the present manuscript is available from the corresponding author on reasonable request. Any proposal will need approval from the ethics committee before sharing any patient data. A signed data transfer agreement will be required if a proposal is approved before data sharing.
