# Supplementary material for: A randomized, double-blind trial comparing the effect of two blood pressure targets on global brain metabolism after out-of-hospital cardiac arrest
Source: Crit Care. 2023 Feb 24;27:73. doi: 10.1186/s13054-023-04376-y (PMC9951410; doi:10.1186/s13054-023-04376-y)
Supplement: Supplementary file 1 — Additional file 1. Supplementary appendix. [file 13054_2023_4376_MOESM1_ESM.docx]

**Supplementary Appendix**

The authors have provided this appendix to give readers additional information about their work.

Supplement to: Mølstrøm S et al. A randomized, double-blind trial comparing the effect of two blood pressure targets on global brain metabolism after out-of-hospital cardiac arrest

**Supplementary Appendix**

Table of Contents

[FUNDING AND ACKNOWLEDGEMENTS 4](#_Toc120635756)

[Funding 4](#_Toc120635757)

[Acknowledgments 4](#_Toc120635758)

[SUPPLEMENTARY METHODS 5](#_Toc120635759)

[BOX-trial design, setting, and population 5](#_Toc120635760)

[Inclusion criteria 6](#_Toc120635761)

[Exclusion criteria 6](#_Toc120635762)

[Details of the intervention 7](#_Toc120635763)

[Post-resuscitation procedure 7](#_Toc120635764)

[Neuromonitoring 8](#_Toc120635765)

[Reasons for withdrawal of life-sustaining therapy 8](#_Toc120635766)

[Outcomes 9](#_Toc120635767)

[Statistical methods 9](#_Toc120635768)

[SUPPLEMENTARY RESULTS 9](#_Toc120635769)

[MAP intervention and Vasopressor-Inotropic Score 9](#_Toc120635770)

[ICU 10](#_Toc120635771)

[Missing data 10](#_Toc120635772)

[Association between cerebral energy metabolism, MAP, and neurological outcome 10](#_Toc120635773)

[JBM variables in relation to critical clinical episodes 10](#_Toc120635774)

[SUPPLEMENTARY FIGURES AND TABLES 12](#_Toc120635775)

[Figure S1. CONSORT flow chart 12](#_Toc120635776)

[Figure S2-S3. Jugular bulb catheter positioning 13](#_Toc120635777)

[Figure S2 13](#_Toc120635778)

[Figure S3 14](#_Toc120635779)

[Figure S4 14](#_Toc120635780)

[Figure S5 15](#_Toc120635781)

[Figure S6 16](#_Toc120635782)

[Figure S7 17](#_Toc120635783)

[Table S1. Post-resuscitation care data 18](#_Toc120635784)

[Table S2. Neurological prognostication 19](#_Toc120635785)

[Table S3. Outcomes and Adverse Events 20](#_Toc120635786)

[Table S4. Baseline characteristics according to neurological outcome at hospital discharge 22](#_Toc120635787)

[Table S5. Jugular bulb microdialysis variables during MAP intervention 23](#_Toc120635788)

[Table S6. Jugular bulb microdialysis variables according to neurological outcome at hospital discharge 24](#_Toc120635789)

[Table S7. Baseline characteristics according to MAP intervention 25](#_Toc120635790)

[References 26](#_Toc120635791)

# FUNDING AND ACKNOWLEDGEMENTS

## Funding

The study has been supported by independent research grants from non-profit organizations or governmental agencies: The University of Southern Denmark, Region of Southern Denmark, Department of Anesthesiology and Intensive Care, Odense, Intensive Conference Hindsgavl, Danish Society of Anesthesiology and Intensive Care Medicine (DASAIM), and the A.P. Moeller Foundation for the Advancement of Medical Science. There was no commercial funding.

## Acknowledgments

We thank patients, relatives, and clinical staff at the Department of Anesthesiology and Intensive Care, Odense University Hospital.

# SUPPLEMENTARY METHODS

## BOX-trial design, setting, and population

The BOX trial was an investigator-initiated, randomized, controlled multi-center study comparing targeted MAP of 63 mmHg vs. 77 mmHg (double-blinded intervention) and liberal vs. restrictive oxygenation targets (open-label 9-10 kPa vs. 13-14 kPa). The patients were randomly allocated to treatment arms in a 1:1:1:1 manner using varying block sizes of 2, 4, or 6 that were stratified according to site. The oxygenation-allocation was concealed until randomization, whereas the MAP intervention remained blinded (trial personnel, trial participants, care providers, outcome assessors) until end-of-follow-up. The trial included 800 adult comatose OHCA-survivors of a presumed cardiac origin in two tertiary heart centers, namely Odense University Hospital (primary hospital for highly specialized cardiac care for 1.3 million citizens) and Copenhagen University Hospital, Rigshospitalet (primary hospital for highly specialized cardiac care for 2.7 million citizens). Inclusion and exclusion criteria for the BOX trial are described below. Per protocol, we included all patients in this sub-study, except for patients randomized to restrictive oxygen target 9-10 kPa (Fig. S1). The primary outcome was combined death or discharge from hospital in a state of Cerebral Performance Category 3 or 4 at 90 days.

## Inclusion criteria

1. Age ≥18 years

2. Out-of-hospital cardiac arrest (OHCA) of presumed cardiac cause

3. Sustained ROSC, defined as ROSC when chest compressions have not been required for 20 consecutive minutes and signs of circulation persist

4. Unconsciousness (GCS <8) after sustained ROSC

5. Target temperature management (TTM)

## Exclusion criteria

1. Conscious patient (GCS ≥ 8)

2. A positive HCG test indicating pregnancy

3. In-hospital cardiac arrest (IHCA)

4. OHCA of presumed non-cardiac cause, e.g., after trauma, dissection/rupture of a major artery, or arrest caused by hypoxia (i.e., drowning, hanging, etc.)

5. Known bleeding diathesis (medically induced coagulopathy does not exclude patient)

6. Suspected or confirmed acute intracranial bleeding

7. Suspected or confirmed acute ischemic stroke

8. Unwitnessed asystole

9. Known limitations in therapy and a do-not-resuscitate order

10. Known disease making 180-day survival unlikely

11. Known pre-arrest CPC score of 3 or 4

12. >240 minutes from ROSC to randomization

13. Systolic blood pressure <80 mmHg despite fluid loading/vasopressor and/or inotropic medication and/or mechanical circulatory support

14. Temperature on admission <30 °C

15. Uncorrected blood glucose at admission <2.5mmol/l

## Details of the intervention

The MAP intervention commenced at randomization and continued as long as the patients needed invasive blood pressure measurements during ICU stay. Patients were randomized to receive monitoring with a module (Phillips M1006B) offset to –10% or a module offset to +10%. The blood pressure modules were modified by a technician (Central Biomedical Engineering Laboratory, Rigshospitalet, Copenhagen, Denmark) with no affiliation to the ICU, enabling blinding of study personnel, care providers, patients, and relatives. Targeting a MAP of 70 mmHg during treatment in both groups will mean a blinded comparison of approximately 63 and 77mmHg, a 20% separation. A randomized, controlled clinical study has validated the method for double-blinded comparison of MAP targets in the ICU setting [13]. Offset modules were used in both MAP groups because the module manufacturer does not support a larger modification than 10% of each module without changing the module software. The modules used in the two trials were identical in appearance, so the caregivers were unaware of which module the patient was on.

## Post-resuscitation procedure

JBM, continuous cardiac output, and near-infrared spectroscopy monitoring were the sole modifications from regional clinical treatment guidelines for comatose OHCA patients. After randomization, comatose patients with sustained ROSC were admitted to the cardiac intensive care unit following OHCA. Immediate angiography and percutaneous coronary intervention (PCI), when indicated, were performed in all resuscitated patients. Targeted temperature management (TTM) commenced at ICU admission targeting 36.0°C for 24 h, followed by controlled rewarming at a rate of 0.5°C/h. Fever control (< 37.5°C) was maintained until 72 h after randomization in patients who remained sedated or comatose.

Hemodynamics were monitored with a radial artery line (MAP) and a pulmonary artery catheter (PAC) inserted through the internal jugular vein under ultrasound guidance. During ICU stay, cardiac index, mixed venous oxygen saturation (SvO_2_), central venous, and pulmonary pressures were measured continuously by PAC (CCOmbo PAC®, Edwards Lifesciences, Irvine, CA, USA) linked to a monitor (Vigilance II®, Edwards Lifesciences). Hemodynamic targets were CVP of 10 to 15 mmHg, urine output >0.5 mL/kg/hour, normal or decreasing lactate, and MAP target of 70 mmHg for as long the patient needed vasopressor and/or inotropes. Norepinephrine was the first-line vasopressor agent, and dopamine was used secondarily. Milrinone was initiated in case of low cardiac output syndrome. A Vasoactive-Inotropic Score (VIS) was calculated as dopamine dose (μg/kg/min) + 100 x epinephrine dose (μg/kg/min) + 10 x milrinone dose (μg/kg/min) + 100 x norepinephrine dose (μg/kg/min) (higher scores indicate a greater degree of support) [1, 2]. Hypovolemia management included volume resuscitation until CVP was at least 10 mmHg and the distensibility index of the inferior vena cava was less than 12 % unless pulmonary edema was clinically apparent. The vasopressor norepinephrine was first-line agent. Blood pressure was not actively lowered using other than analgosedation during TTM to meet the MAP target of 70 mmHg.

## Neuromonitoring

Intravenous MD catheters (CMA 67 IV, MDialysis AB, Stockholm, Sweden) were placed in the jugular vein. The dominant jugular vein was accessed by retrograde insertion of an MD catheter (130 mm) through a 16 G intravenous catheter, with the tip located in the jugular bulb under ultrasound guidance. The ideal positioning of the MD catheter tip corresponds to the anatomical landmark at the level of the mastoid. In accordance with previous studies, the precise positioning of the jugular bulb catheter tip was confirmed on cranial computed tomography (CT) scan (figures S2-S3). The catheters were perfused from an MD pump (CMA 106, MDialysis AB, Stockholm, Sweden) MD at a flow rate of 0.3 μL/min with Ringers Acetate and Dalteparin Sodium (25 IU/mL). Hourly samples of energy-related metabolites (lactate, pyruvate, glucose, glutamate, glycerol) were collected in microvials and analyzed using enzymatic photometric techniques (Iscus, MDialysis AB, Stockholm, Sweden). The relative recovery in cerebral tissue was about 70% for the variables studied with this perfusion rate. The relative recovery is expected to be considerably higher when placed in venous blood. In a pilot study, the accuracy of the MD catheter was assessed by exploring the correlation and agreement between systemic blood lactate (Lac_sys_) and MD arterial lactate (Lac_MD-Art_). A highly significant correlation with r=0.73 and coefficient at 0.82 [0.75-0.89] (p<0.0001) was obtained. Bland-Altman statistics showed an average bias for Lac_sys_ of 0.18 mM higher than Lac_MD-Art_, with the 95 % limits of agreement ranging from −0.75 to 1.11. In this JBM study, systemic lactate replaced the invasive MD arterial lactate monitoring as a reference [3].

Standard 30-minute EEGs were evaluated by a clinical neurophysiologist blinded to clinical information. The EEG with the most malignant pattern was assessed in the case of multiple EEGs. However, in the presence of EEGs recorded with and without sedation, the EEG without sedation with the most malignant pattern was used. Electrographic seizure activity, suppression, burst-suppression, low voltage, continuity of background activity, periodic discharges, periodic or rhythmic patterns, and electrographic reactivity were recorded. Based on these findings, EEGs were classified as highly malignant, malignant, or benign [4].

## Reasons for withdrawal of life-sustaining therapy

In comatose patients with a Glasgow Motor Score of ≤3 at ≥72 h from ROSC, in the absence of confounders, a poor outcome was considered when two or more of the following predictors were present: no pupillary and corneal reflexes at ≥72 h, bilaterally absent N20 SSEP wave at ≥24 h, highly malignant EEG at ≥24 h (suppressed background ± periodic discharges or burst-suppression), status myoclonus ≤72 h (continuous and generalized myoclonus persisting for 30 minutes or more), or a diffuse and extensive anoxic injury on brain CT. If a decision on WLST was made, the time point and the main reasons for withdrawing life-supporting therapies were recorded.

## Outcomes

Neurological outcome was assessed at hospital discharge and 90 days after OHCA according to the Cerebral Performance Category (CPC) scale: CPC scores of 1-2 are considered 'favorable' outcomes, and a CPC 3-5 'unfavorable' outcomes. Experienced neurologists performing the CPC assessment determined the score based on telephone interviews with patients or medical records. The pre-defined serious adverse events (SAE) related to interventions were bleeding, sepsis, arrhythmia, and microdialysis catheter complications.

## Statistical methods

For patient characteristics, results were expressed as counts and proportions, median with IQR or mean±SD, as appropriate. Unpaired t-tests or Mann–Whitney U-tests were used for unpaired comparisons of numerical variables. Chi-square or Fischer's exact test was applied to examine differences between categorical variables. Dynamic changes of MD variables were analyzed longitudinally, applying a linear mixed model including fixed effects for treatment group and time period and a fixed time-treatment interaction. Repeated measurements of MD variables on the same patient over time were taken into account by including a random intercept for each patient. The mixed model fitting procedure handled missing values, assumed to be missing at random. Overall, missing MD data rates were less than 5%. Missing data imputation was not used to replace missing data points with substituted values.

# SUPPLEMENTARY RESULTS

## MAP intervention and Vasopressor-Inotropic Score

In the low MAP group, the median Vasoactive-Inotropic Score (VIS) during the first 24 h after admission was significantly higher (low MAP: 18 (IQR 11–28), high MAP: 8 (IQR 4–14 μg/kg/min), p=0.0001), see Table S1 and Figure S4. The difference in VIS score was mainly caused by significantly higher doses of Norepinephrine (low MAP: 0.17 (IQR 0.09–0.27 μg/kg/min), high MAP: 0.09 (IQR 0.05–0.14 μg/kg/min), p=0.006), Table S1. Based on a previous pilot trial, we expected significantly higher vasopressor-doses in the high MAP group [5, 6]. Still, the opposite was observed during the first 24 h caused by several factors, including the presence of a higher percentage of shock and organ hypoperfusion in the low MAP group (Table 1), reduced vasopressor requirements in the high MAP group (seven patients without vasopressor need) and small sample size. During the intervention, no differences in median dopamine dose between MAP groups were observed.

## ICU

The median ICU length of stay for low and high MAP groups were 98 [69-141] and 91 [64-164] hours, and related sedation time for the groups was 32 [25-44] and 36 [26-42] hours. During ICU stay, there was no significant difference in cardiac output, mixed venous saturations, and systemic lactate (Table S1). Reduced global metabolic demand (TTM and analog-sedation) during the first ICU days was illustrated by high and stable levels of mixed venous saturations associated with low cardiac output.

Additional regulators of cerebral blood flow remained within therapeutic range with no difference in PaO_2_ and PaCO_2_ at any time points between MAP groups. Results for neurological prognostication are presented in Table S2.

## Missing data

Two patients in the high MAP group and one patient in the low MAP group were excluded due to failed MD monitoring, otherwise, only a few JBM catheters were associated with temporary malfunction due to clotting. During monitoring time, missing microdialysis data rates were less than 5% except for glutamate (11.6%) and characterized as missing completely at random (lactate: 4.93%, pyruvate: 5.3%, glycerol: 6.3%, glucose 4.93%). No patients were lost to follow-up.

## Association between cerebral energy metabolism, MAP, and neurological outcome

Based on the biochemical definitions, approximately 75% of all patients exhibited ongoing secondary ischemia during 22% of total MD monitoring, irrespective of MAP allocation (Table S3). In almost all patients, biochemical signs of mitochondrial dysfunction were detected 31% and 35% of the time in low MAP and high MAP groups (Table S3), respectively. No significant difference between MAP groups related to the extent of ischemia (p=0.88) and mitochondrial dysfunction (p=0.54) was observed when applying mixed-effects logistic regression.

Almost all patients with unfavorable outcomes displayed mitochondrial dysfunction signs during 32% of total MD monitoring time versus 90% in 37% (n=21) of the time in the CPC 1-2 group, respectively. In patients with unfavorable outcome, 73% of the patients exhibited ongoing secondary ischemia during 23% of total MD monitoring. 78% of patients with favorable outcome displayed signs of ischemia 19% of the time. No significant difference between CPC groups related to the extent of ischemia and mitochondrial dysfunction was observed when applying mixed-effects logistic regression.

## JBM variables in relation to critical clinical episodes

Among patients (n=14) with malignant and highly malignant EEG patterns, no differences in JBM levels were observed. Few patients in outcome groups showed minimal transient signs of cerebral desaturation with rSO2 <50% in <2% of the total INVOS monitoring period. No correlations between JBM-verified ischemic periods and cerebral desaturation were observed.

# SUPPLEMENTARY FIGURES AND TABLES

## Figure S1. CONSORT flow chart


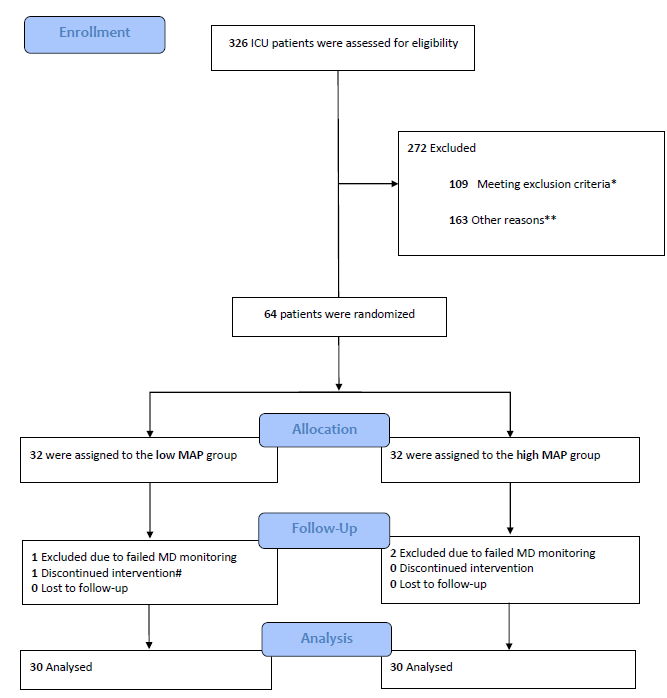


Text Figure 1. Screened, excluded, and included patients in the study. * Exclusion criteria were: OHCA with presumed non cardiac cause (asphyxia, trauma, massive bleeding, aortic dissection) (n=26), conscious patients (GCS ≥ 8) (n=25), suspected or confirmed acute intracranial bleeding (n=5), > 240 minutes from ROSC to randomization (n=6), refractory shock (n=12), unwitnessed asystole (n=13), known pre-arrest cerebral performance category (CPC) score of 3 or 4 (n=5), in-hospital cardiac arrest (n=17). ** Other reasons: participating in other studies (n=131), logistics (n=26), and the trial staff was not informed about the patient (n=6). # Hemodynamic instability and mechanical assist device (Impella). Abbreviations: ICU, intensive care unit; MAP, mean arterial pressure; MD, microdialysis.

## Figure S2-S3. Jugular bulb catheter positioning

The correct positioning of the jugular bulb catheter tip (red arrow) was confirmed on cranial computed tomography (CT) scan; see figures S2 and S3 for examples.

### Figure S2


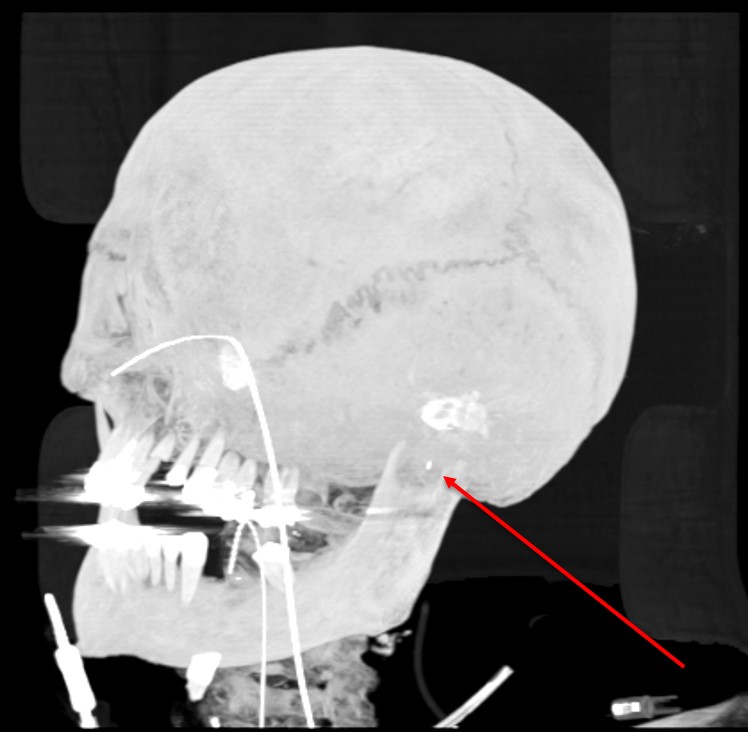


Text Figure S2. CT scan with 3D reconstruction documented a correct positioning of the jugular bulb catheter tip (red arrow). The image has been downloaded from the GE Web Pacs database with permission to use in the article.

### Figure S3


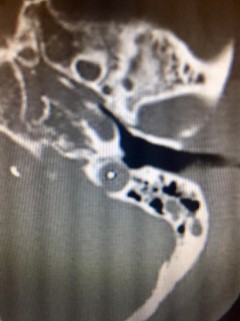


Text Figure S3. CT scan in the axial plane documented a correct positioning of the jugular bulb catheter tip (red arrow). The image has been downloaded from the GE Web Pacs database with permission to use in the article.

## Figure S4


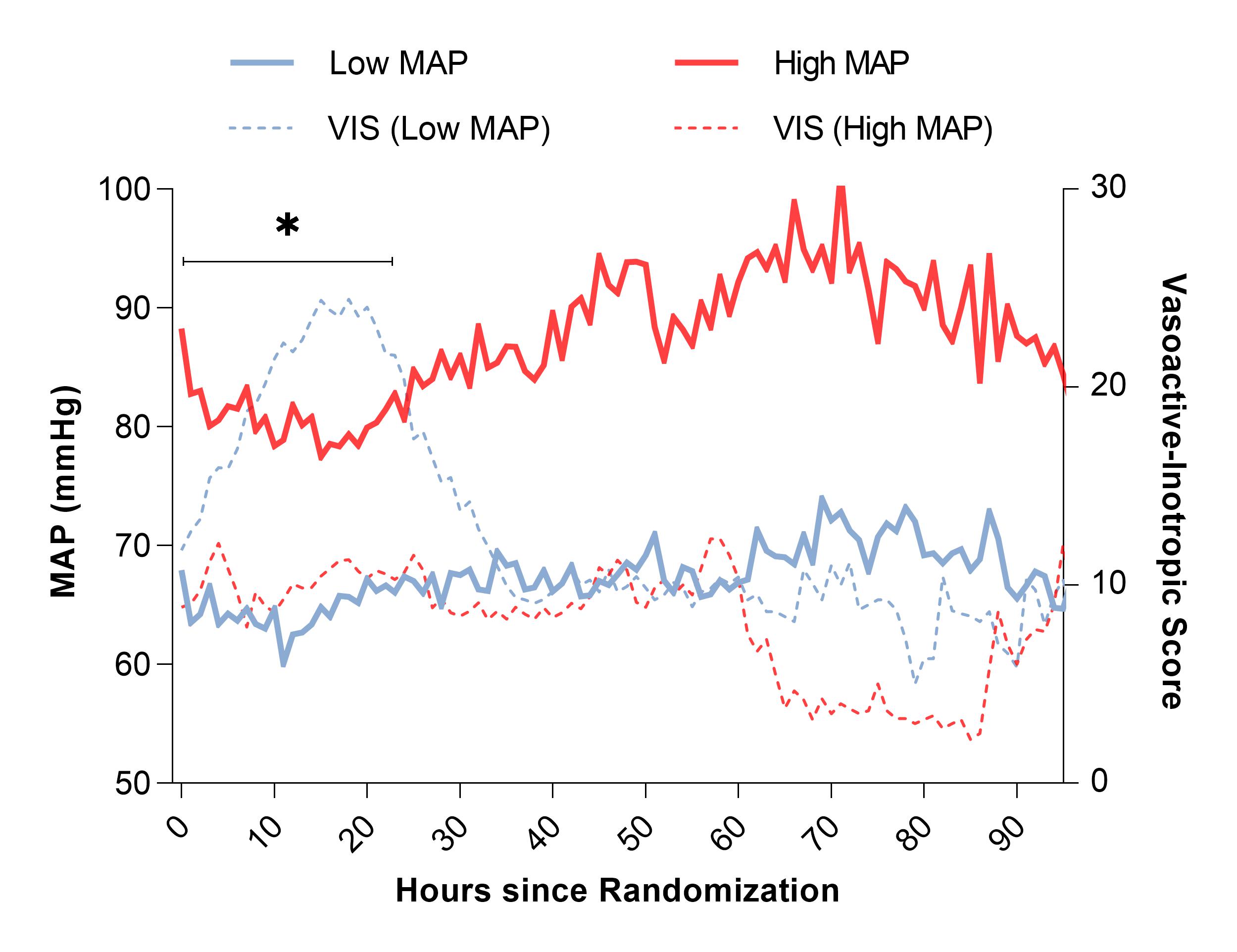


Text Figure S4. Mean arterial pressure and calculated Vasoactive-Inotropic Score (VIS) during the intervention period. The MAP and dotted VIS curves show the means. In the low MAP group, the VIS score during the first 24h after admission was significantly higher (p=0.0001). Data not stratified for neurological outcome.

## Figure S5


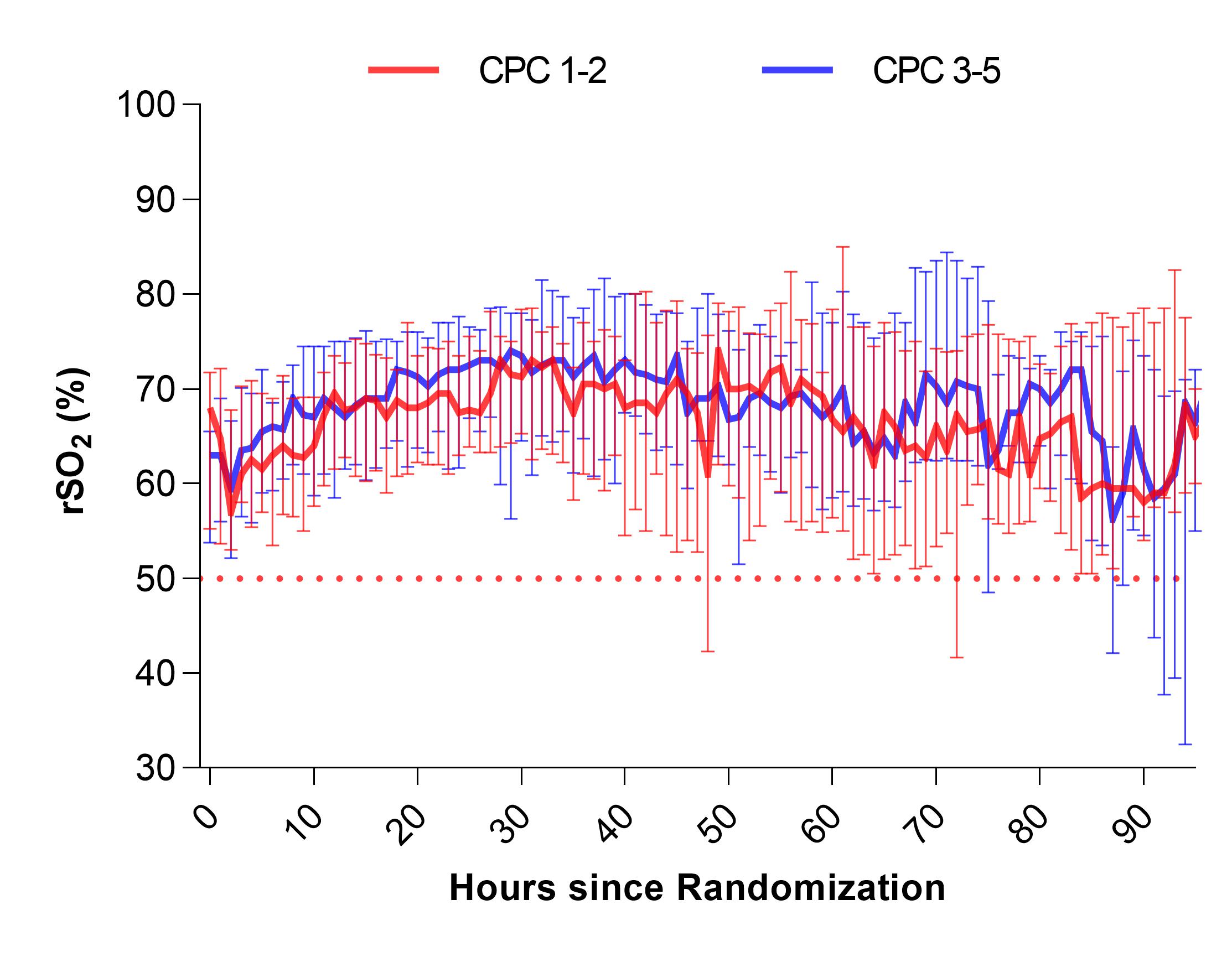


Text Figure S5. Median (IQR). Regional cerebral oxygen saturation (rSO_2_) during post-resuscitation care in patients with favorable (CPC 1-2, n=23) and unfavorable (CPC 3-5, n=37) outcome. No significant differences between outcome groups were observed. Data not stratified for MAP intervention. The dotted red line indicates cerebral desaturation threshold rSO_2_ <50 %.

## Figure S6


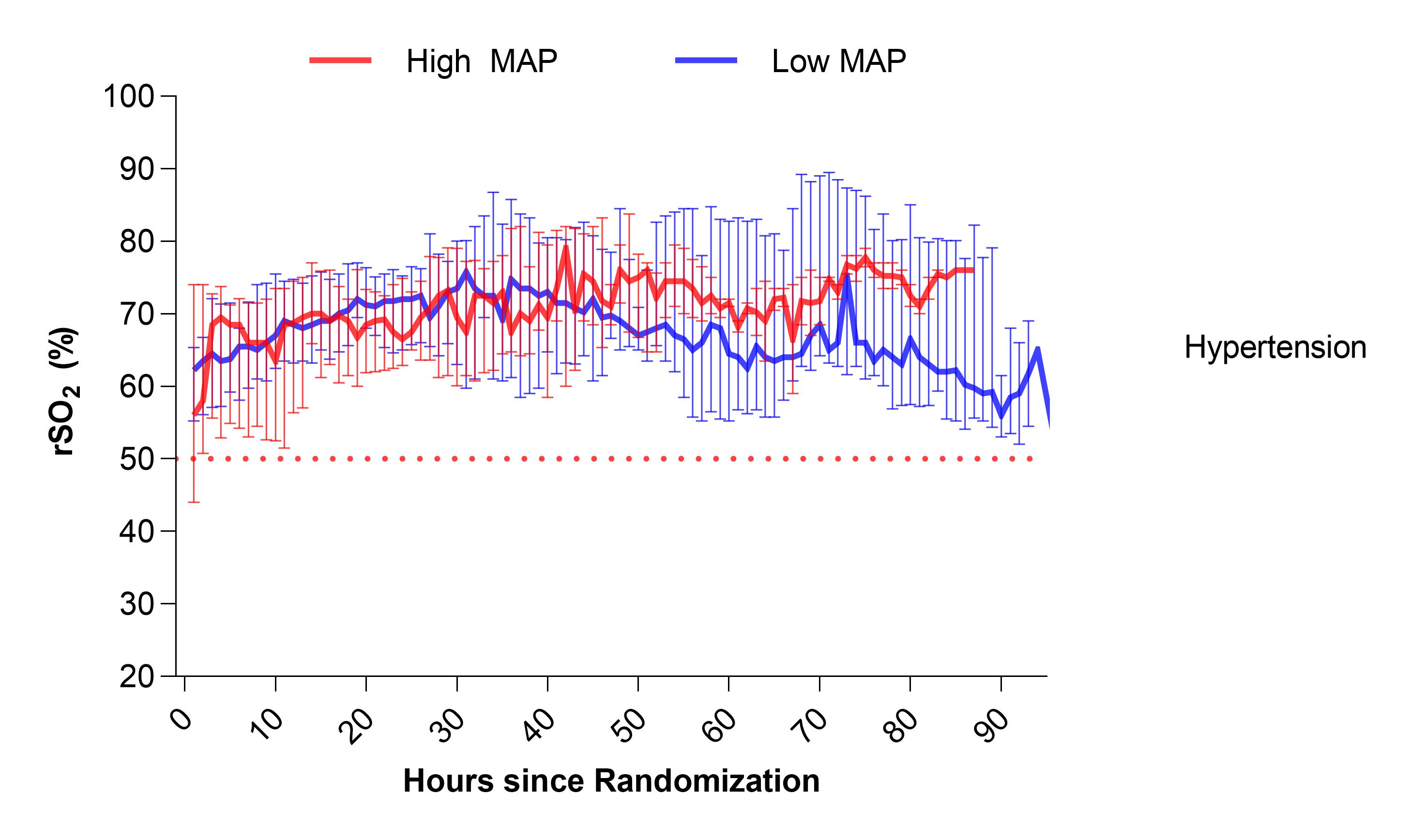


Text Figure S6. Median (IQR). Regional cerebral oxygen saturation (rSO_2_) during post-resuscitation care in patients with chronic hypertension randomized to low (n=19) or high MAP (n=13). No significant differences between MAP groups were observed. Data not stratified for neurological outcome. The dotted red line indicates cerebral desaturation threshold rSO_2_ <50 %.

## Figure S7


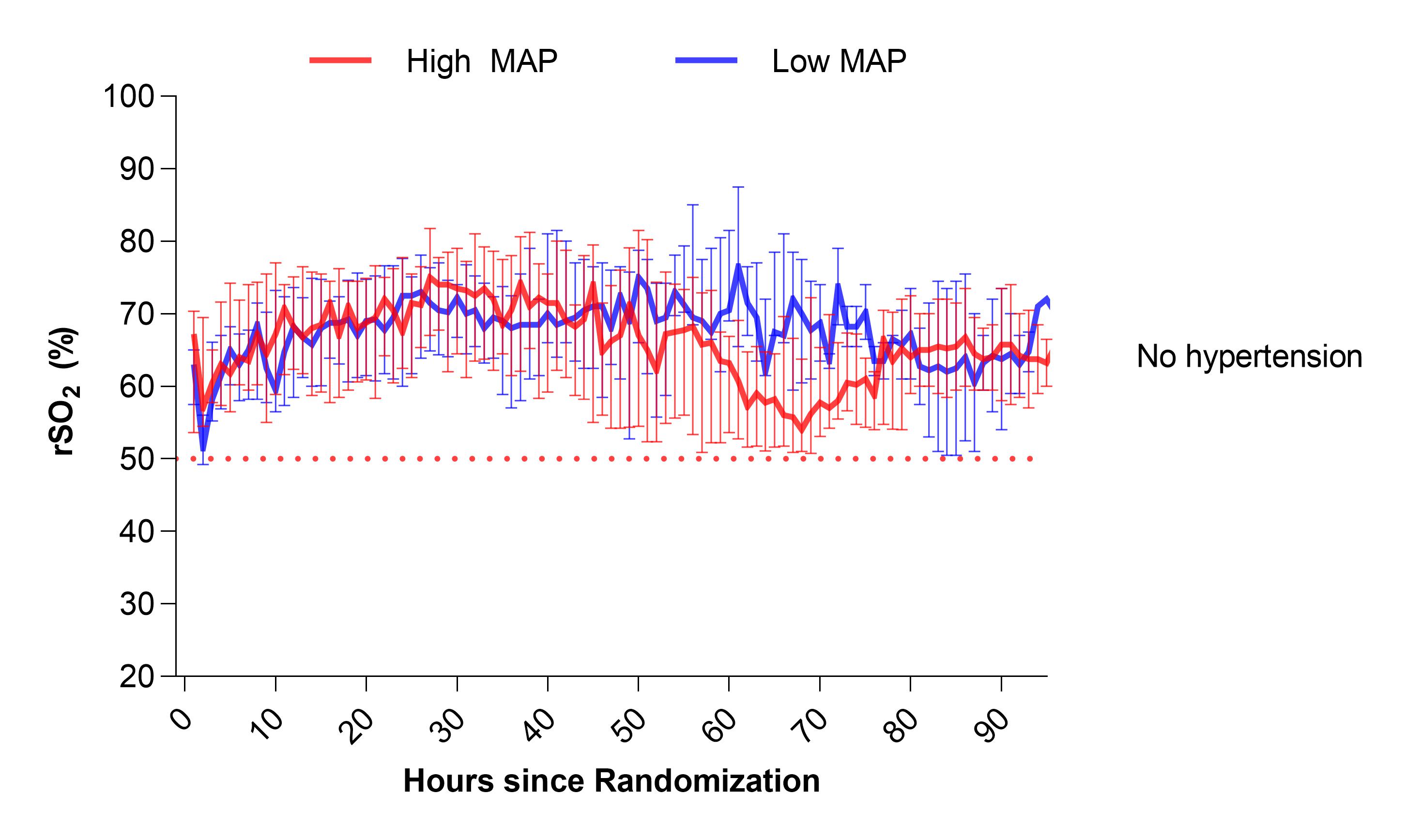


Text Figure S7. Median (IQR). Regional cerebral oxygen saturation (rSO_2_) during post-resuscitation care in patients without hypertension randomized to low (n=10) or high MAP (n=13). No significant differences between MAP groups were observed. Data not stratified for neurological outcome. The dotted red line indicates cerebral desaturation threshold rSO_2_ <50 %.

## Table S1. Post-resuscitation care data

| ICU monitoring | Low MAP  (n = 30) | High MAP  (n = 30) |
| --- | --- | --- |
| Hemodynamics |  |  |
| MAP – mmHg  Day 1  Day 2  Day 3  Day 4 | 64±6  67±8  68±9  70±8 | **80±9**  **86±12**  **90±13**  **91±15** |
| CI – l/min/m^2^  Day 1  Day 2  Day 3  Day 4 | 2.4±0.9  3.1±0.8  3.1±0.7  3.3±0.7 | 2.2±0.5  2.8±0.8  3.1±0.9  3.1±0.9 |
| SvO_2_ – %  Day 1  Day 2  Day 3  Day 4 | 73±8  72±7  70±7  71±8 | 70±7  71±6  71±7  72±9 |
| Average serum lactate - mmol/liter  Day 1  Day 2  Day 3  Day 4 | 1.9±1.4  1.6±0.8  1.4±0.7  1.1±0.4 | 1.7±1.9  1.4±0.8  1.3±0.5  1.2±0.3 |
| Average NE infusion (µg/kg/min), median (IQR)  Day 1  Day 2  Day 3  Day 4 | **0.17 [0.09-0.27]**  0.12 [0.05-0.18]  0.06 [0.04-0.09]  0.07 [0.05-0.15] | 0.09 [0.05-0.14]  0.07 [0.03-0.12]  0.04 [0.02-0.10]  0.03 [0.02-0.05] |
| Average VIS, median (IQR)  Day 1  Day 2  Day 3  Day 4 | **18 [11-28]**  9 [3-18]  6 [4-11]  7 [3-13] | 8 [4-14]  6 [3-12]  6 [2-10]  4 [2-6] |
| PaO_2_ – kPa  Day 1  Day 2  Day 3  Day 4 | 14.4±3.1  13.8±2.8  13.5±2.8  14.2±2.5 | 14.0±3.0  13.5±2.4  13.2±2.9  13.1±2.5 |
| PaCo_2_ – kPa  Day 1  Day 2  Day 3  Day 4 | 5.5±0.8  5.6±0.7  5.4±0.8  5.5±0.8 | 5.3±0.8  5.5±0.7  5.4±0.7  5.6±0.5 |

Text Table S1. Plus-minus values are means ± SD. Bold text indicates a statistically significant difference with a p-value less than 0.05. n = number of patients. Abbreviations: MAP, Mean arterial pressure; CI, Cardiac index; SvO_2_, mixed venous saturations; NE, norepinephrine; VIS, Vasoactive-Inotropic Score. P<0.05 is highlighted in bold.

## Table S2. Neurological prognostication

| Prognostication – no./total no. (%) | Low MAP  (n = 30) | High MAP  (n = 30) |
| --- | --- | --- |
| Prognostication | 10/30 (33%) | 11/30 (37%) |
| Time from OHCA to prognostication (hours) | 76 [58-87] | 93 [79-108] |
| CT, generalized edema | 3/10 (30%) | 6/11 (55%) |
| EEG seizure | 1/10 (10%) | 3/11 (27%) |
| EEG classification*  Benign  Malignant  Highly malignant | 2/10 (20%)  6/10 (60%)  2/10 (20%) | 3/11 (27%)  4/11 (36%)  4/11 (36%) |
| SSEP, bilaterally absent N20-respons | 2/10 (20%) | 2/11 (18%) |
| Recommendation  Continue care  Withdraw care | 5/10 /50%)  5/10 (50%) | 6/11 (55%)  5/11 (45%) |

Text Table S2. N = number of patients. Median (IQR). Abbreviations: OHCA, out-of-hospital cardiac arrest; CT, computed tomography; SSEP, somatosensory-evoked potentials; EEG, electroencephalogram; *EEG; benign (absence of malignant features), malignant (malignant periodic or rhythmic patterns, malignant background or unreactive EEG), highly malignant (suppressed background without discharges, suppressed background with continuous periodic discharges; burst-suppression). A higher MAP of approximately 77 mmHg did not significantly affect the EEG findings or brain edema compared with a lower MAP level of 63 mmHg.

Table S3. Outcomes and Adverse Events

| **Outcomes or Events** | **Low MAP**  **(n = 30)** | **High MAP**  **(n = 30)** |
| --- | --- | --- |
| Primary outcome: |  |  |
| LP ratio within the first 24 hours – median (IQR)  Day 1  Day 2  Day 3  Day 4 | 19 [16-31]  15 [12-20]  15 [13-21]  14 [12-18] | 23 [16-33]  16 [13-21]  15 [12-20]  17 [12-24] |
| Secondary outcomes: |  | |
| Jugular bulb microdialysis – median (IQR) |  |  |
| Time from ROSC to MD analysis (min) | 255 [236-300] | 268 [202-345] |
| Microdialysis time (hours) | 65 [47-91] | 63 [45-89] |
| Metabolic pattern  Ischemia*  Mitochondrial dysfunction** | 21.4% (375 h), n=23  31.2% (547 h), n=28 | 22.3% (357 h), n=22  34.8% (557 h), n=29 |
| INVOS - (rSO_2_ %) - median (IQR)  Day 1  Day 2  Day 3  Day 4  Cerebral desaturation - % of total INVOS  monitoring*** | 69 [62-74]  72 [64-77]  68 [63-80]  64 [60-70]  1.3% (18 h), n=4 | 68 [60-74]  72 [64-78]  70 [63-75]  73 [69-76]  1.5% (18 h), n=6 |
| Median NSE at 48 h (IQR) — μg/liter **** | 15 [10-27] | 12 [9-28] |
| Mortality 90 days after OHCA - no./total no. (%) | 12/30 (40%) | 8/30 (27%) |
| Time to death (days) – median (IQR) | 6 [4-9] | 6 [3-8] |
| Location of death  ICU  Ward | 5/12 (42%)  7/12 (58%) | 4/8 (50%)  4/8 (50%) |
| Cause of death  Cerebral  Hemodynamic | 10/12 (84%)  2/12 (16%) | 6/8 (75%)  2/8 (25%) |
| Neurologic function at hospital discharge  no./total no. (%) |  |  |
| Good, CPC score 1-2 | 8/30 (27%) | 15/30 (50%) |
| CPC Category^#^  1  2  3  4  5 | 2/30 (7%)  6/30 (20%)  10/30 (33%)  -  12/30 (40%) | 5/30 (17%)  10/30 (33%)  6/30 (20%)  1/30 (3%)  8/30 (27%) |
| Serious Adverse Events^¤^ – no./total no. (%) |  |  |
| Bleeding  Sepsis  Arrhythmia  Microdialysis catheter complications | 0/0 (0%)  1/30 (3%)  4/30 (13%)  0/0 (0%) | 0/0 (0%)  2/30 (6%)  3/30 (10%)  0/0 (0%) |

Text Table S3. Median (IQR). Bold text indicates a statistically significant difference with a p-value <0.05. N = number of patients. Abbreviations: MD, microdialysis; ICU, intensive care unit; INVOS, In-Vivo Optical Spectroscopy (regional oxygen saturation); NSE, neuron-specific enolase; OHCA, out-of-hospital cardiac arrest; CPC, Cerebral Performance Category. *Microdialysis verified ischemia defined as LPR >16 and pyruvate <70 µmol/l. Percentage (%) of total MD monitoring during ICU stay. **Microdialysis verified mitochondrial dysfunction defined as LPR >16 and pyruvate >70 µmol/l. Percentage (%) of total MD monitoring time during ICU stay. ***Cerebral desaturation defined as rSO_2_ <50%. **** Reference values for neuron-specific enolase range from 0 to 16.3 μg per liter. Levels at 48 hours were available for 39 patients (20 in the low MAP group and 19 in the high MAP group).

^#^CPC score: 1, alert, able to work and lead a normal life; 2, moderate cerebral disability and sufficient cerebral function for part-time work; 3, severe cerebral disability, dependent on others, and impaired brain function; 4, coma and vegetative state; 5, dead or certified brain dead. ^¤^Uncontrolled bleeding (>1 unit of blood/10 kg/1h), bleeding causing fatality, intracerebral bleeding, septic shock, and arrhythmia resulting in hemodynamic compromise. Microdialysis catheter complications; carotid artery puncture, pneumothorax, venous hematoma, catheter-related thrombosis, or infection. No significant differences between groups were observed.

## Table S4. Baseline characteristics according to neurological outcome at hospital discharge

| **Characteristic** | **CPC 1-2**  **N = 23** | **CPC 3-5**  **N = 37** |
| --- | --- | --- |
| **Demographic characteristics** |  |  |
| Age – years | 62±16 | 69±11 |
| Male sex – no. (%) | 21 (91) | 31 (84) |
| **Medical history – no. (%)** |  |  |
| Chronic heart failure | 1 (4) | 9 (24) |
| Ischemic heart disease | 7 (30) | 11 (30) |
| Arterial hypertension | 11 (48) | 23 (62) |
| Previous stroke | 1 (4) | 2 (5) |
| Diabetes mellitus | 3(13) | 8 (22) |
| Previous percutaneous coronary intervention | 4(17) | 7 (19) |
| **Neurological function before cardiac arrest** |  |  |
| Normal, CPC score 1* | 22 (96) | 33 (89) |
| Some disabilities, CPC score 2 | 1 (4) | 4 (11) |
| **Characteristics of the cardiac arrest** |  |  |
| Witnessed cardiac arrest – no. (%) | 21 (91) | 31 (86) |
| Bystander performed CPR – no. (%) | 22 (96) | 32 (89) |
| First monitored rhythm – no. (%) |  |  |
| Shockable rhythm | 20 (87) | 24 (65) |
| Time from cardiac arrest to event – min |  |  |
| Start of basic life support, median (IQR) | 1 [1-3] | 1 [1-5] |
| Start of advanced life support, median (IQR)  Return of spontaneous circulation, median (IQR) | 8 [5-11]  15 [10-29] | 10 [5-14]  20 [12-25] |
| **Clinical characteristics on admission** |  |  |
| First measured body temperature – °C | 35.1±1.3 | 35.4±1.1 |
| Glascow Coma Scale score §, median (IQR) | 3 [3-3] | 3 [3-3] |
| Pupillary reflex present – no. (%) | 17 (74) | 19 (51) |
| Serum pH  Serum lactate – mmol/liter | 7.25±0.12  4.7±4.2 | 7.23±0.15  5.5±4.1 |
| Shock – no. (%) ¶ | 4 (17) | 11 (30) |
| ST-segment elevation myocardial infarction – no. (%) | 12 (52) | 14 (38) |

Text Table S4. Plus-minus values are means ± SD. Abbreviations: CPC, Cerebral Performance Category; AMI, acute myocardial infarction; CPR, cardiopulmonary resuscitation; IQR, interquartile range. * CPC score: 1, alert, able to work and lead a normal life; 2, moderate cerebral disability and sufficient cerebral function for part-time work; 3, severe cerebral disability, dependent on others, and impaired brain function; 4, coma and vegetative state; 5, dead or certified brain dead. CPC scores of 1-2 are considered 'favorable' outcomes, and CPC 3-5 'unfavorable' outcomes. § Scores on the Glasgow Coma Scale range from 3 to 15, with lower scores indicating reduced levels of consciousness. ¶ Shock was defined as a systolic blood pressure of less than 90 mmHg for more than 30 min or end-organ hypoperfusion (cool extremities, confusion, urine output <0.5 ml/kg per hour, lactate >2.5 mmol/l). No significant differences between groups were observed.

## Table S5. Jugular bulb microdialysis variables during MAP intervention

| Hours since Randomization | LP ratio | | Lactate mM | | Pyruvate µM | |  |
| --- | --- | --- | --- | --- | --- | --- | --- |
|  | Low MAP | High MAP | Low MAP | High MAP | Low MAP | High MAP |  |
| 12 h | 24 [18-38] | 25 [17-38] | 2.5 [1.6-3.4] | 2.0 [1.5-3.1] | 85 [51-155] | 83 [61-109] |  |
| 24 h | 18 [15-24] | 21 [15-29] | 2.1 [1.4-2.8] | 1.8 [1.3-2.6] | 104 [64-162] | 82 [64-117] |  |
| 36 h | 15 [13-20] | 16 [13-22] | 1.7 [1.2-2.5] | 1.7 [1.2-2.5] | 106 [77-148] | 93 [73-131] |  |
| 48 h | 14 [12-19] | 15 [13-20] | 1.4 [1.1-1.8] | 1.6 [1.3-2.4] | 98 [66-132] | 101 [87-145] |  |
| 60 h | 15 [12-21] | 15 [12-19] | 1.4 [1.0-2.0] | 1.4 [1.1-1.9] | 92 [77-116] | 96 [69-127] |  |
| 72 h | 15 [13-21] | 15 [12-21] | 1.4 [1.0-2.0] | 1.4 [1.1-2.3] | 95 [67-122] | 96 [76-118] |  |
| 84 h | 15 [12-19] | 17 [12-25] | 1.4 [1.0-2.0] | 1.5 [1.3-1.9] | 91 [68-124] | 85 [65-114] |  |
| 96 h | 15 [11-19] | 17 [13-20] | 1.1 [0.7-1.9] | 1.4 [1.3-1.6] | 73 [55-114] | 99 [65-113] |  |
|  | | | | | | |  |
| Hours since Randomization | Glucose mM | | Glycerol µM | | Glutamate µM | |  |
|  | Low MAP | High MAP | Low MAP | High MAP | Low MAP | High MAP |  |
| 12 h | 6.5 [4.4-7.9] | 5.7 [4.3-6.9] | 215 [153-291] | 162 [116-236] | 51 [33-71] | 56 [41-80] |  |
| 24 h | 6.3 [5.3-7.7] | 6.1 [4.8-7.0] | **221 [164-340]** | 179 [142-232] | 52 [27-73] | 50 [33-70] |  |
| 36 h | 7.0 [6.1-8.0] | 6.5 [5.3-8.0] | **174 [128-262]** | 133 [103-175] | 56 [42-74] | 50 [37-78] |  |
| 48 h | 7.0 [5.9-8.0] | 7.2 [5.7-8.2] | 114 [90-155] | 105 [72-142] | 50 [38-65] | 45 [34-63] |  |
| 60 h | 6.8 [6.0-7.9] | 6.8 [5.3-8.2] | 105 [74-143] | 70 [55-118] | 50 [38-67] | 47 [35-58] |  |
| 72 h | 6.8 [6.0-8.4] | 7.9 [6.3-8.9] | 86 [64-121] | 82 [55- 112] | 40 [31-60] | 53 [42-59] |  |
| 84 h | 7.1 [6.3-9.3] | 7.9 [6.7-9.0] | 77 [58-113] | 81 [61- 99] | 32 [25-50] | 55 [40-63] |  |
| 96 h | 8.2 [6.4-10.1] | 8.3 [7.4-9.6] | 79 [61-117] | 82 [60- 110] | 28 [17-40] | 54 [56-61] |  |
|  | | | | | | |  |

Text Table S5. Data are expressed as median (interquartile range). LP ratio: lactate/pyruvate ratio. The difference between time-averaged means of MD variables in patients randomized to low versus high MAP group was insignificant during post-resuscitation care except for glycerol when using mixed-effects models. Data not stratified for neurological outcome. Hourly samples were analyzed separately, and total samples were averaged for intervals of 12 hours. P<0.05 is highlighted in bold.

## Table S6. Jugular bulb microdialysis variables according to neurological outcome at hospital discharge

| Time from Randomization | LP ratio | | Lactate mM | | Pyruvate µM | |  |
| --- | --- | --- | --- | --- | --- | --- | --- |
|  | CPC 1-2 | CPC 3-5 | CPC 1-2 | CPC 3-5 | CPC 1-2 | CPC 3-5 |  |
| 12 h | 23 [17-32] | 26 [18-43] | 1.8 [1.3-2.7] | **2.5 [1.7-3.5]** | 79 [61-97] | **97 [53-156]** |  |
| 24 h | 19 [15-25] | 19 [14-28] | 1.6 [1.2-2.4] | **2.1 [1.4-3.1]** | 78 [66-109] | **104 [63-168]** |  |
| 36 h | 16 [14-20] | 15 [13-22] | 1.6 [1.2-2.1] | 1.9 [1.2-2.7] | 89 [73-120] | **108 [77-159]** |  |
| 48 h | 15 [12-18] | 15 [13-20] | 1.4 [1.2-1.9] | 1.6 [1.2-2.3] | 96 [68-130] | 104 [77-143] |  |
| 60 h | 15 [12-18] | 15 [12-22] | 1.4 [1.0-1.7] | 1.4 [1.1-2.1] | 93 [68-118] | 96 [76-122] |  |
| 72 h | 14 [12-21] | 15 [13-21] | 1.4 [1.1-2.3] | 1.5 [1.0-2.0] | 99 [81-113] | 92 [62-127] |  |
| 84 h | 17 [13-23] | 15 [12-20] | 1.5 [1.3-1.8] | 1.5 [1.0-2.0] | 87 [74-111] | 90 [64-120] |  |
| 96 h | 20 [15-23] | 14 [12-19] | 1.3 [1.2-1.6] | 1.3 [0.9-1.9] | 87 [57-106] | 81 [61-115] |  |
|  | | | | | | |  |
| Time from Randomization | Glucose mM | | Glycerol µM | | Glutamate µM | |  |
|  | CPC 1-2 | CPC 3-5 | CPC 1-2 | CPC 3-5 | CPC 1-2 | CPC 3-5 |  |
| 12 h | 5.8 [4.2-7.4] | 6.1 [4.5-7.7] | 174 [122-246] | 193 [144-288] | 59 [45-79] | 50 [35-70] |  |
| 24 h | 6.1 [5.0-7.2] | 6.2 [5.1-7.7] | 173 [141-227] | **216 [160-316]** | 57 [37-75] | 46 [27-67] |  |
| 36 h | 6.8 [5.7-8.1] | 6.8 [5.9-8.0] | 133 [106-172] | **168 [120-255]** | 57 [44-77] | 50 [35-72] |  |
| 48 h | 7.2 [5.6-8.2] | 7.0 [6.0-8.0] | 114 [93-147] | 104 [76-151] | 49 [37-64] | 48 [34-63] |  |
| 60 h | 7.7 [5.5-8.5] | 6.5 [5.8-7.7] | 83 [60-121] | 92 [64-134] | 52 [36-59] | 46 [38-66] |  |
| 72 h | 8.5 [7.5-9.3] | 6.6 [5.5-8.1] | 74 [53-126] | 85 [63-110] | 53 [40-62] | 44 [29- 57] |  |
| 84 h | 8.4 [7.3-9.4] | 7.2 [6.0-8.6] | 68 [52-89] | 85 [63-109] | 61 [41-66] | 38 [25-53] |  |
| 96 h | 9.3 [8.2-10.4] | 8.0 [6.4-9.6] | 69 [47-82] | 87 [64-127] | 54 [46-58] | 29 [17-40] |  |
|  | | | | | | |  |

Text Table S6. Data are expressed as median (interquartile range). LP ratio: lactate/pyruvate ratio. Jugular bulb microdialysis parameters during post-resuscitation care in patients with favorable outcome (CPC 1-2, n=23) compared to patients with unfavorable outcome (CPC 3-5, n=37). The difference between time-averaged means of MD variables between outcome groups was assessed using mixed-effects models. Data not stratified for MAP group. Hourly samples were analyzed separately, and total samples were averaged for intervals of 12 hours. P<0.05 is highlighted in bold.

## Table S7. Baseline characteristics according to MAP intervention

| **Characteristic** | **Low MAP**  **N = 30** | **High MAP**  **N = 30** | **P value** |
| --- | --- | --- | --- |
| **Demographic characteristics** |  |  |  |
| Age – years | 69±12 | 63±14 | 0.066 |
| Male sex – no. (%) | 28 (93) | 24 (80) | 0.254 |
| **Medical history – no. (%)** |  |  |  |
| Chronic heart failure | 8 (27) | 3 (10) | 0.080 |
| Ischemic heart disease | 11 (37) | 7 (23) | 0.399 |
| Arterial hypertension | 20 (67) | 14 (47) | 0.192 |
| Previous stroke | 3 (10) | 0 (0) | 0.237 |
| Diabetes mellitus | 7 (23) | 4 (13) | 0.506 |
| Previous percutaneous coronary intervention ^a^ | 6 (20) | 5 (17) | 1.000 |
| **Neurological function before cardiac arrest** |  |  |  |
| Normal, CPC score 1* | 26 (87) | 29 (97) | 0.353 |
| Some disability, CPC score 2 | 4 (13) | 1 (3) |  |
| **Characteristics of the cardiac arrest** |  |  |  |
| Witnessed cardiac arrest – no. (%) ^b^ | 27 (90) | 26 (87) | 1.000 |
| Bystander performed CPR – no. (%) | 26 (87) | 29 (97) | 0.195 |
| First monitored rhythm – no. (%) |  |  |  |
| Shockable rhythm | 22 (73) | 22 (73) | 1.000 |
| Time from cardiac arrest to event – min |  |  |  |
| Start of basic life support, median (IQR) | 2 [1-5] | 1 [1-3] | 0.358 |
| Start of advanced life support, median (IQR)  Return of spontaneous circulation, median (IQR) | 8 [5-10]  16 [10-23] | 10 [6-16]  20 [12-32] | 0.301  0.514 |
| **Clinical characteristics on admission** |  |  |  |
| First measured body temperature – °C | 35.5±1.0 | 35.1±1.3 | 0.186 |
| Glascow Coma Scale score §, median (IQR) | 3 [3-3] | 3 [3-3] | NA |
| Pupillary reflex present – no. (%) | 17 (57) | 19 (63) | 0.792 |
| Serum pH ^c^  Serum lactate – mmol/liter | 7.26±0.12  5.1±4.1 | 7.23±0.15  5.2±4.3 | 0.421  0.895 |
| Shock – no. (%) ¶ | 11 (36) | 8 (26) | 0.580 |
| ST-segment elevation myocardial infarction – no. (%) | 8 (27) | 18 (60) | 0.018 |

Text Table S7. Plus-minus values are means ± SD. Abbreviations: CPC, Cerebral Performance Category; AMI, acute myocardial infarction; CPR, cardiopulmonary resuscitation; IQR, interquartile range. * CPC score: 1, alert, able to work and lead a normal life; 2, moderate cerebral disability and sufficient cerebral function for part-time work; 3, severe cerebral disability, dependent on others, and impaired brain function; 4, coma and vegetative state; 5, dead or certified brain dead. § Scores on the Glasgow Coma Scale range from 3 to 15, with lower scores indicating reduced levels of consciousness. ¶ Shock was defined as a systolic blood pressure <90 mmHg for more than 30 min or end-organ hypoperfusion (cool extremities, confusion, urine output <0.5 ml/kg per hour, lactate >2.5 mmol/l).

^a^ Data missing for one patients, ^b^ Data missing for one patients, ^c^ Data missing for two patients.

# References

1. Vallabhajosyula S, Jentzer JC, Kotecha AA, Murphree DH, Jr., Barreto EF, Khanna AK, Iyer VN, (2018) Development and performance of a novel vasopressor-driven mortality prediction model in septic shock. Ann Intensive Care 8: 112

2. Goradia S, Sardaneh AA, Narayan SW, Penm J, Patanwala AE, (2021) Vasopressor dose equivalence: A scoping review and suggested formula. Journal of critical care 61: 233-240

3. Molstrom S, Nielsen TH, Nordstrom CH, Forsse A, Moller S, Veno S, Mamaev D, Tencer T, Schmidt H, Toft P, (2021) Bedside microdialysis for detection of early brain injury after out-of-hospital cardiac arrest. Sci Rep 11: 15871

4. Westhall E, Rossetti AO, van Rootselaar AF, Wesenberg Kjaer T, Horn J, Ullen S, Friberg H, Nielsen N, Rosen I, Aneman A, Erlinge D, Gasche Y, Hassager C, Hovdenes J, Kjaergaard J, Kuiper M, Pellis T, Stammet P, Wanscher M, Wetterslev J, Wise MP, Cronberg T, investigators TT-t, (2016) Standardized EEG interpretation accurately predicts prognosis after cardiac arrest. Neurology 86: 1482-1490

5. Grand J, Meyer AS, Kjaergaard J, Wiberg S, Thomsen JH, Frydland M, Ostrowski SR, Johansson PI, Hassager C, (2020) A randomised double-blind pilot trial comparing a mean arterial pressure target of 65 mm Hg versus 72 mm Hg after out-of-hospital cardiac arrest. European heart journal Acute cardiovascular care 9: S100-S109

6. Grand J, Meyer ASP, Hassager C, Schmidt H, Moller JE, Kjaergaard J, (2018) Validation and Clinical Evaluation of a Method for Double-Blinded Blood Pressure Target Investigation in Intensive Care Medicine. Critical care medicine 46: 1626-1633
